# Systemic responses to intravenous rAAV administration in boys with Duchenne muscular dystrophy

**DOI:** 10.3389/fmmed.2026.1953867

**Published:** 2026-09-23

**Authors:** Brooke E. Harris, Brooke M. Seldin, Sam C. Towne, Orinda R. Hobson, Eli Zerpa, Victor Prima, H. Lee Sweeney, David W. Hammers

**Affiliations:** 1 Department of Pharmacology & Therapeutics, University of Florida College of Medicine, Gainesville, FL, United States; 2 Myology Institute, University of Florida, Gainesville, FL, United States; 3 Department of Molecular Genetics and Microbiology, University of Florida College of Medicine, Gainesville, FL, United States

**Keywords:** adeno-associated virus, complement activation, Duchenne muscular dystrophy, immune system, interferon response genes, micro-dystrophin, SomaScan

## Abstract

**Introduction:**

Systemic gene therapies utilizing recombinant adeno-associated virus (rAAV) are in the clinic for the treatment of rare monogenic diseases. This includes delandistrogene moxeparvovec for Duchenne muscular dystrophy (DMD), which uses AAVrh74 vector to deliver a micro-dystrophin transgene to striated muscle. While there have been cases of transformative benefits incurred from rAAV therapies, serious adverse events and mortalities have occurred. This study aimed to investigate short-term rAAV dynamics and long-term systemic responses to intravenous rAAV in the phase 1/2a delandistrogene moxeparvovec clinical trial (NCT03375164), which represents the most promising cases of micro-dystrophin gene therapy for DMD.

**Methods:**

Plasma and urine specimens from NCT03375164 were analyzed for rAAV using digital PCR and immunoblotting. The integrity and transduction competency of specimen-derived rAAV was evaluated *in vitro*. Systemic responses to rAAV treatment were investigated using the SomaScan proteomics platform. Preclinical antiviral responses to rAAV were assayed by real-time PCR in the livers of D2. *mdx* mice 1 week following systemic treatment with rAAV.

**Results:**

Digital PCR revealed high rAAV content in the plasma at Day 1 following treatment, which substantially reduced by Day 7. Urinary rAAV content was low and variable in all samples tested. The majority of detected vector genomes were packaged in AAVrh74 particles in these specimens, and the vector contained in Day 1 plasma samples was confirmed to be transduction competent. SomaScan data revealed highly significant changes in 99 circulating proteins, which include markers associated with immune activation, interferon responses, complement pathways, and organ injury. Most traditional markers of muscle damage were not changed across the time course, with the limitation that no healthy or untreated DMD samples were analyzed. Features of antiviral and interferon-associated responses were identified in D2. *mdx* mice.

**Conclusion:**

Systemic rAAV treatments result in high levels of circulating and transduction-competent vector early after treatment in DMD boys and result in wide-spread activation of immune-associated markers. These findings will provide insights for future investigational study design and efforts to improve safety of rAAV gene therapies.

## Introduction

There are currently five FDA-approved gene therapies that utilize intravenous administration of recombinant adeno-associated viral (rAAV) vectors to deliver therapeutic transgenes for the treatment of lethal monogenic diseases. This includes delandistrogene moxeparvovec for the treatment of Duchenne muscular dystrophy [DMD; (Mendell et al., 2020)], an X-linked pediatric onset muscular dystrophy caused by loss of the muscle stabilizing protein, dystrophin (Hoffman et al., 1987; Petrof et al., 1993). The strategy to treat DMD *via* delandistrogene moxeparvovec entails the packaging of the highly truncated ΔR4-R23ΔCT micro-dystrophin transgene (Harper et al., 2002) in an AAVrh74 serotype vector to enable dystrophin replacement therapy in striated muscles of affected individuals (Mendell et al., 2020). The goal of this treatment is to transform DMD to a slower progressing disease that resembles milder forms of muscular dystrophy (England et al., 1990). While these state-of-the-art molecular therapeutics offer the potential for transformative outcomes for individuals affected by these devastating diseases, the high viral doses required to achieve adequate treatment transduction have led to serious, and even fatal, adverse events during their clinical implementation (Lek et al., 2023a; Lek et al., 2023b; Potter et al., 2025; Maurizi et al., 2026). Due to these inherent risks associated with intravenous rAAV therapies, a greater understanding of the systemic responses to high-dose rAAV in humans is needed to improve the safety of rAAV gene therapy implementation in DMD and other monogenic diseases.

Systemic administration of high rAAV doses has been associated with a spectrum of adverse events (AEs), ranging from transient flu-like symptoms to severe immune-mediated toxicities, including hepatotoxicity, thrombotic microangiopathy, complement activation, and fatal outcomes (Maurizi et al., 2026). These events are thought to be driven largely by innate and humoral immune recognition of both the viral capsid and vector genome (Lek et al., 2023a; Keeler et al., 2025), which can trigger robust inflammatory cascades and organ stress responses. These risks are exacerbated by high doses used to achieve sufficient tissue transduction. Together, these observations highlight a need to better understand how systemic responses may affect both the safety and effectiveness of rAAV gene therapies.

The purpose of this study was to investigate rAAV dynamics and underlying systemic responses to rAAV administration in young DMD boys in the absence of overt SAEs using plasma and urine samples procured during the course of the phase 1/2a delandistrogene moxeparvovec clinical trial [NCT03375164; (Mendell et al., 2020)]. Molecular analysis of these samples identified high levels of packaged and transduction competent rAAV early after treatment, significant declines in plasma vector levels 1 week following treatment, and low and variable evidence of urinary shedding. Additionally, SomaScan data reveal broad proteomic changes in plasma spanning muscle disease markers, immune activation, and complement activation. These findings provide a detailed picture of acute and sustained systemic responses to rAAV administration in DMD boys.

## Materials and methods

### Study approval

Plasma and urine samples were procured from the University of Florida Biorepository under Institutional Review Board protocol #201802247. Animal procedures were approved and conducted in accordance with the University of Florida IACUC.

### Animal procedures

Male D2. *mdx* mice (Jax# 013141) received control or rAAV (2 × 10^14^ vg/kg of AAVrh10. CB6PI. EGFP; n = 4) at 6 months of age *via* intravenous (IV) tail vein injection. Tissues were harvested 1 week following treatment and were stored at −80° C until analysis.

### Cell culture

*In vitro* transduction competency was assayed using the AC16 human cardiomyocyte cell line (ATCC #CRL-3568) maintained in DMEM F-12 media (Gibco # 10565–018) supplemented with 12.5% FBS (Gibco #A38401-01) and 1% Pen/Strep (Gibco #15140–122). Cells were plated at a density of 40,000 cells per well in a 96-well plate 24 hours prior to administration of ∼1 × 10^10^ vg of rAAV [estimated by digital (d)PCR] diluted in 50 µL of serum-free media supplemented with 1.25% BSA (Thermo Scientific Cat No J10857-22). Alongside cells treated with rAAV, a negative control (serum-free media only) and a rAAVrh10 positive control (Progen #66V101; 98.8% homology with AAVrh74) were also tested. Following 2 h of rAAV exposure, cells were washed three times with serum-free media, and the plate was incubated for 24 h in full media. Cells were lysed with proteinase K lysis buffer [13.1 mM sodium deoxycholate (Sigma Aldrich D675-256); 7.5 mM sodium dodecyl sulfate (Sigma Aldrich L3771-1 KG); 1% Polysorbate 20 (Fisher Scientific BP337-500); ≥180 U Proteinase K (Invitrogen #AM2546)], transferred to PCR tubes, and run on a thermocycler with the following conditions: 37 °C for 1 h, 55 °C for 2 h, 95 °C for 30 min, and storage at 4 °C. Vector genome/diploid genome content in cell digests were assessed using real-time PCR, as previously described (Hammers et al., 2020a) using the primers for AAV ITR2 (forward: 5′- GGA​ACC​CCT​AGT​GAT​GGA​GTT -3′; reverse: 5′- CG GCCTCAGTGAGCGA -3′) to quantify vector genomes and genomic regions in human *RPP30* locus (forward: 5′- GCT​CAG​CTC​TTC​AGG​AAA​CA -3′; reverse: 5′- ACA​ATA​CAG​CCA​CAG​AAA​GAGA -3′) to quantify diploid genomes.

### Digital PCR

AAV vector genomes were isolated from plasma and urine samples using the DNeasy Blood and Tissue Kit (Qiagen #69504) following the manufacturer’s protocol, using a volume of 100 µL of sample. The concentration of AAV vector genomes in urine and plasma samples (n = 6 technical replicates) was determined *via* absolute quantification using the QIAcuity dPCR system. Isolated DNA samples were diluted in buffer containing 10X GeneAmp PCR Buffer (Applied Biosystems #N8080006), MgCl_2_ Solution (included with Applied Biosystems #N8080006), 0.05% Poloxamer 188 (Gibco #24040–032), 0.002 mg/mL Sheared Salmon Sperm (Invitrogen #AM9680) and nuclease free-water. A diluted sample volume of 5 µL was combined with dPCR master mix containing 4X QIAcuity Probe Mastermix (Qiagen PN 250102), 0.25 Units/µL HpaII (Thermo Scientific #ER0511), 0.9 µM of each AAV ITR2 primers (forward: 5′- GGA​ACC​CCT​AGT​GAT​GGA​GTT -3′; reverse: 5′- CGGCCTCAGTGAGCGA -3′) primers, 0.25 µM of FAM-labeled ITR2 probe (5′- CAC​TCC​CTC​TCT​GCG​CGC​TCG-3′), and nuclease-free water. Samples were loaded into wells of an 8.5 k 96-well nanoplate (Qiagen #250021) for the QIAcuity dPCR system. The following thermal cycling conditions were applied for dPCR: initial denaturation at 95 °C for 2 min, amplification with 40 cycles at 95 °C for 15 s followed by 55 °C for 30 s. Partitioning and absolute quantification analysis was performed in the QIAcuity software (version 3.2) which yielded absolute copies/mL of AAV vector genomes in the original sample volume. Samples that were determined to be <LLOQ were ran at the following dilutions: 1:1, 1:2, and 1:4.

For evaluation of vector genome content only from intact particles in samples, 50 µL of a DNase cocktail containing 5 units of DNase I (Promega #M610 A), DNase 10X Reaction Buffer (Promega #M198 A), 0.1% Poloxamer 188 (Gibco #24040–032) and nuclease-free water was added to 50 µL of urine or plasma. After these samples were incubated at 37 °C for 30 min, 5 µL of Stop Solution (Promega #M199 A) was added to the reaction mix and incubated at 65 °C for 10 min. Following DNase treatment, these samples were immediately processed using the Qiagen DNeasy Blood and Tissue Kit following the manufacturer’s protocol, and concentration of AAV vector genomes determined *via* dPCR as described above.

### Reverse transcription PCR

Reverse transcription PCR (RT-PCR) of control and rAAV-treated mouse livers was performed as previously described (Hammers et al., 2020b) using mouse-specific primers for *Mx1* (forward: 5′- AAG​AGC​TCT​GTG​CTG​GAA​GC -3′; reverse: 5′- GGG​CCC​ATA​GCC​TAG​CAA​AG -3′), *Ifit3* (forward: 5′- GTG​GAC​TGA​GAT​TTC​TGA​ACT​GC -3′; reverse: 5′- GAT​TCC​CGG​TTG​ACC​TCA​C TC -3′), *Ifnb* (forward: 5′- CTC​CAC​CAG​CAG​ACA​GTG​TT -3′; reverse: 5′- CCAGGCGTAGCTGTT GTACT -3′), *Mavs* (forward: 5′- CAG​CTA​GTG​ACC​AGG​ATC​G -3′; reverse: 5′- CGGCGCTGGAGATTATTGAAG -3′), and *Rpl32* (forward: 5′-GGG​TGC​GGA​GAA​GGT​TCA​A-3′; reverse: 5′-GCAATCTCA​GCA​CAG​TAA​GAT​TTG​T-3′). Relative gene expression quantification was performed using the ΔΔCt method with *Rpl32* as the normalization gene.

### SDS-PAGE and immunoblotting

SDS-PAGE and immunoblotting were performed as previously described (Hammers et al., 2017; Hammers et al., 2020b) using the anti-AAV VP1/VP2/VP3 (Progen #65158; 1:100) primary antibody. AAVrh10 empty capsid was run as a positive control (Progen #66V100), serially diluted (1:10, 1:100, 1:1,000) from a starting concentration of 6.5 × 10^12^ vp/mL. Plasma samples were diluted 1:20 and then 2.5 µL loaded per lane. Urine samples were loaded as is with 5 µg loaded per lane. The Licor C-Digit system was used to image and quantify enhanced chemiluminescent signals. Equal sample loading was confirmed by Ponceau Red staining.

### SomaScan proteomics

Plasma samples were shipped to SomaLogic for proteomic analysis using the SomaScan platform (V4, 5 k; SomaLogic, Inc.), which uses DNA-based aptamers (SOMAmer reagents) for protein-capture to report specific epitope content as relative fluorescence units (Gold et al., 2010). Assay data were accessed using the SomaDataIO R package (https://somalogic.github.io/SomaDataIO/) for downstream analyses. The full dataset is found in the supplemental data file.

### Statistical analysis

Statistical analysis was performed using one-way ANOVA (Tukey *post hoc* tests; α = 0.05) and two-tailed paired or unpaired Student’s T-tests (α = 0.05), where appropriate. SomaScan data were analyzed by repeated measures ANOVA (Tukey *post hoc* tests; α = 0.05) using JMP statistical software (SAS). Data are presented as means and individual data points in violin plots, mean ± SEM with individual data points in line plots, or as volcano plots.

## Results

### rAAVrh74 dynamics in plasma and urine

For the purposes of investigating vector dynamics and systemic responses following intravenous administration of rAAV in humans, we procured biorepository deposited plasma and urine specimens collected during the first year of NCT03375164, an open-label phase 1/2a trial evaluating delandistrogene moxeparvovec in young (>4 to <8 years old; n = 4) boys with DMD (Mendell et al., 2020). As detailed in the initial report (Mendell et al., 2020), participants were required to have an anti-AAVrh74 binding-antibody titer ≤1:400 and were screened for baseline hepatic and hematologic values–including GGT (10.0 U/L), ALT (556.0 U/L), and platelets (420.3 K/mm^3^). Participants received 2.0 × 10^14^ vg/kg of rAAVrh74. MHCK7. micro-dystrophin *via* peripheral limb vein infusion, were on a weekend prednisolone regimen during the course of the study and received daily prednisolone during the first 30 days post-infusion [(Mendell et al., 2020); Figure 1]. These 4 boys exhibited no SAEs, effective micro-dystrophin expression in limb muscle, and multiyear functional improvements (Mendell et al., 2020; Mendell et al., 2024; Mendell et al., 2026). Furthermore, magnetic resonance imaging performed on three of the participants revealed reduced fat replacement and T_2_ signatures of inflammation in lower limb muscles relative to natural history controls (Willcocks et al., 2021). AAVrh74 IgG-specific B-cell responses were also found to be substantially elevated in all individuals by Day 14 (Mendell et al., 2020). In light of the safety profiles and functional outcomes recently reported for delandistrogene moxeparvovec [EMBARK; NCT05096221; (Mendell et al., 2025)] and fordadistrogene movaparvovec [CIFFREO; NCT04281485; (Muntoni et al., 2026)] in phase 3 trials, these 4 individuals are among the best representatives for successful micro-dystrophin gene therapy for the treatment of DMD.

**FIGURE 1:**
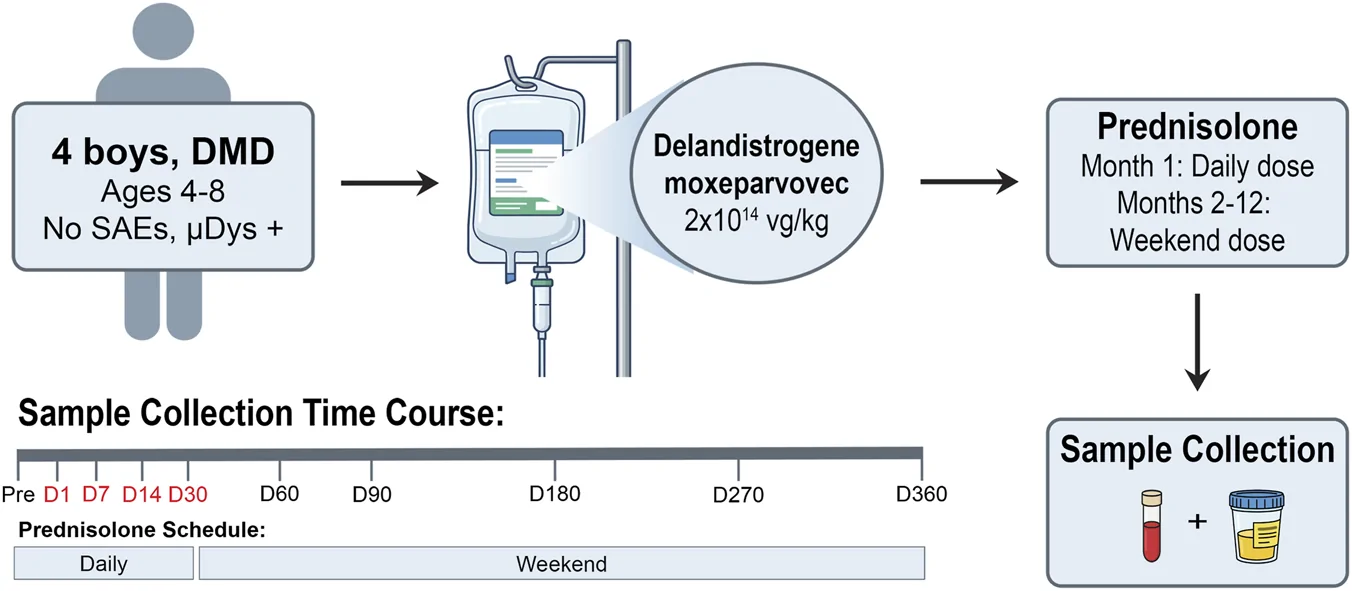
Study Design for clinical samples procured. Blood and urine samples were procured from the phase 1/2a delandistrogene moxeparvovec clinical trial (NCT03375164). In summary, 4 boys with Duchenne muscular dystrophy (DMD; ages >4 and <8 years) received a single intravenous infusion of AAVrh74. MHCK7. micro-dystrophin (µDys; dose of 2 × 10^14^ vg/kg). Prednisolone was administered daily during month 1 and on a weekend only schedule during months 2–12. Blood and urine samples were collected at baseline (Pre-Tx) and on days 1, 7, 14, 30, 90, 180, 270, and 360 post-treatment.

To investigate aspects of vector dynamics following intravenous rAAV treatment, we utilized dPCR and immunoblotting to quantify the content of rAAV genomes and capsid in the procured biospecimens, respectively. Viral genome levels from the plasma of the 4 boys, over the time course of baseline (Pre-Tx), 1 day post-treatment (Day 1), and 7 days post-treatment (Day 7), were found to be the highest at Day 1 (≥1 × 10^12^ vg/mL) and drop substantially at Day 7 (≤1 × 10^9^ vg/mL; Figure 2A). Urine samples over the same time course, had genome titers that were low and variable among participants. Two participants had viral genome levels below the lower limit of quantification (<LLOQ; estimated to be 2,700 vg/mL) across all time points. One participant had detectable levels at Day 1 (5 × 10^5^ vg/mL), that then dropped < LLOQ by Day 7, and one participant had detectable genome titers at both Days 1 and 7 (6 × 10^6^ and 1 × 10^6^ vg/mL, respectively; Figure 2A; urine samples were not normalized to creatinine or specific gravity). Individual participant values for vector genome titers in plasma and urine can be found in Supplementary Table S1.

**FIGURE 2:**
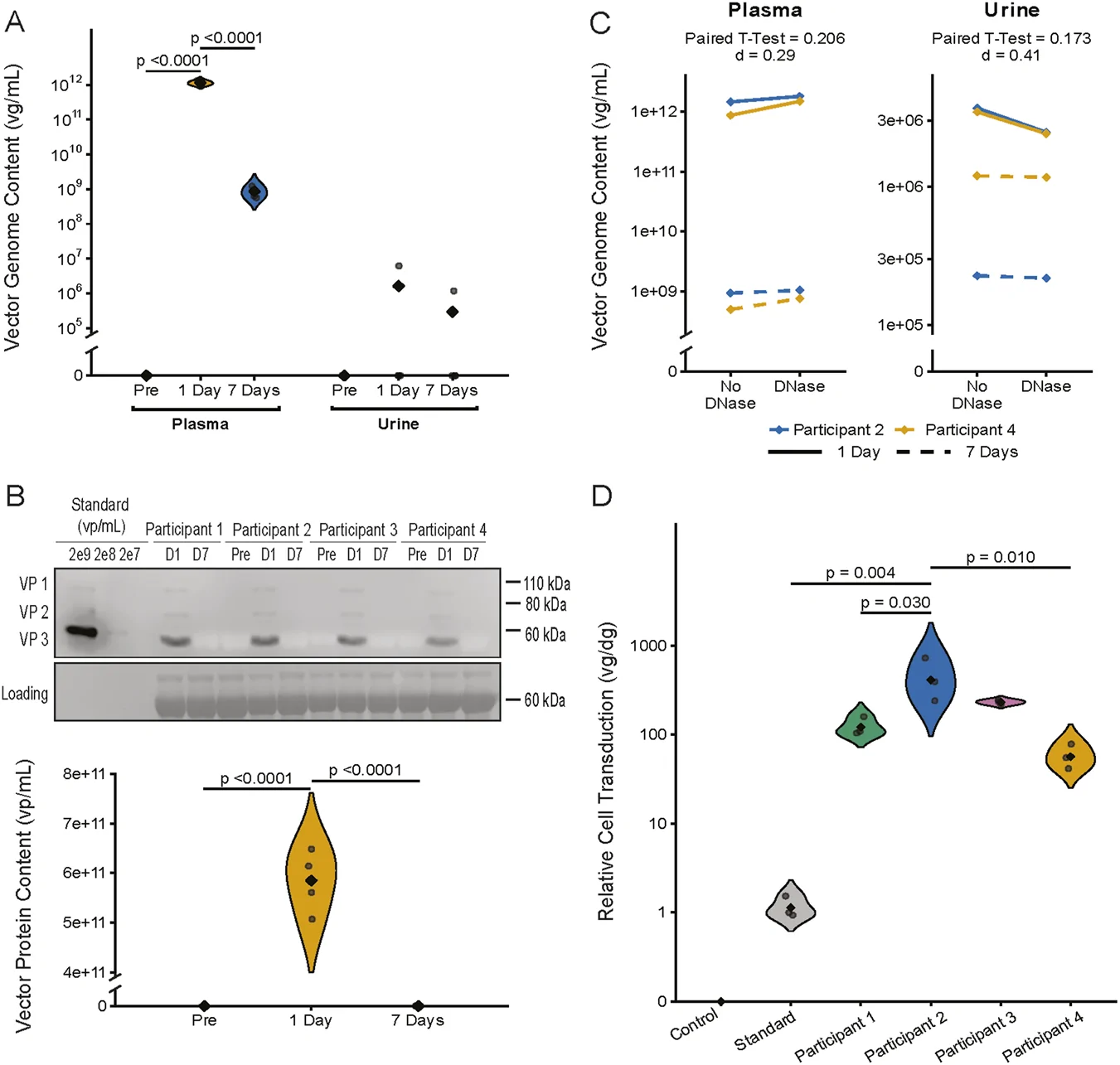
Dynamics of rAAV in plasma and urine of DMD boys. To assess the dynamics of rAAV in plasma and urine biospecimens sampled at baseline (Pre-Tx), Day 1, and Day 7 following treatment with 2 × 10^14^ vg/kg of AAVrh74. MHCK7. micro-dystrophin **(A)** plasma vector genome content was measured by digital PCR (values below limit of detection are indicated as zero), **(B)** AAV capsid protein levels in plasma and estimated viral particles were measured by immunoblotting (estimated from empty capsid standard signal; values below limit of detection are shown as zero), **(C)** Integrity of rAAV in plasma and urine was determined by dPCR of total and DNase-treated specimens for participants 2 and 4 at Day 1 and Day 7, **(D)** Transduction competency of circulating plasma rAAV was confirmed at Day 1 (normalized to an rAAV standard). Data are displayed as **(A,B and D)** violin plots with individual values indicated by circles and means indicated by black diamonds (pre-screening: n = 3; Day 1 and Day 7: n = 4), and **(C)** a line plot **(n = 4)**, with the Y-axis in log_10_ scale. Data were analyzed using **(A,B and D)** one-way ANOVA with *post hoc* Tukey analysis (α = 0.05; significant p-values are indicated) or **(C)** paired t-test (α = 0.05; p-value and effect size indicated).

Immunoblotting for AAV capsid levels of the plasma and urine were consistent with these findings, as AAV capsid VP proteins were strongly detected in Day 1 plasma (Figure 2B). The rAAV concentration (vector particles/mL) in plasma samples were interpolated from detectable standard concentrations and agree with dPCR findings, demonstrating consistency of rAAV genome and proteome components analyzed from biobanked plasma samples. The Day 7 plasma samples and all urine samples across all participants were below the limit of detection of this assay (Figure 2B; Supplementary Figure S1). Individual participant values for estimated vector proteins in plasma and urine are also reported in Supplementary Table S1.

Recent evidence suggests that rAAVs are capable of spontaneous genome ejection at body temperature (37 °C) conditions (Gliwa et al., 2025), which could result in a situation where released vector genomes and empty rAAV capsids accumulate in circulation. To determine if such a phenomenon occurs in systemic treatments of rAAVrh74, we investigated dPCR titers in plasma and urine samples with and without pretreatment with DNase. Since DNase treatment degrades unprotected genomes, the ratio reflects the proportion of packaged *versus* unpackaged genomes contained in these biospecimens. As shown in Figure 2C, viral genomes quantified in plasma samples procured at both Day 1 and Day 7 did not demonstrate significant differences with DNase pretreatment. While the corresponding urine samples also did not show significant changes with DNase pretreatment, Day 1 urine specimens did trend towards lower vector genome content in DNase pre-treated samples. This suggests that rAAV genome ejection is not a prominent feature of systemic rAAVrh74-based therapies, however, some unpackaged vector genomes may be excreted in urine at early timepoints following rAAV infusion.

To further investigate rAAV integrity in procured biospecimens, we also tested the transduction competency of viral particles in Day 1 plasma samples using AC16 human cardiomyocytes. Following a 24-h incubation in media supplemented with the volume of plasma to achieve ∼2 × 10^8^ vg/µL (as determined using dPCR data), real-time PCR analysis of cell lysates for vector genome content revealed comparable transduction levels across all Day 1 participant samples (Figure 2D). Viral content from Day 7 plasma and all urine samples were too low to adequately test vector integrity after longer circulation durations and excretion using this assay. These data agree with those from the DNase pretreatment experiment and confirm that rAAVrh74 vector retains integrity and transduction competency for at least 1 day following systemic treatment in DMD boys.

### Systemic responses to intravenous rAAV treatment

In parallel with these studies, aliquots of all available plasma samples were submitted for SomaScan aptamer-based proteomic profiling [(Gold et al., 2010; Hathout et al., 2015); Pre-Tx to Day 270: n = 4; Day 360: n = 2] to investigate systemic biomarkers associated with high-dose rAAV administration and treatment efficacy. Of the >5,000 protein targets analyzed in this assay, repeated measures analysis using data from Pre-Tx to Day 270 identified 287 targets demonstrating significant changes across the time course [false discovery rate (FDR) p-value of <0.01; Figure 3A]. A higher scrutiny threshold (FDR p-value <0.001) identified 99 significantly changed targets (Figure 3B; Supplementary Table S2), which were enriched for genes associated with infection/immune responses, iron regulation, extracellular matrix (ECM) production, and organism development, as determined by Reactome, KEGG, and GO pathway enrichment analyses (Table 1). Overall, these data reveal that potent activation of immune-associated pathways dominate the systemic responses to rAAV administration, even in the absence of overt clinical phenotypes.

**FIGURE 3:**
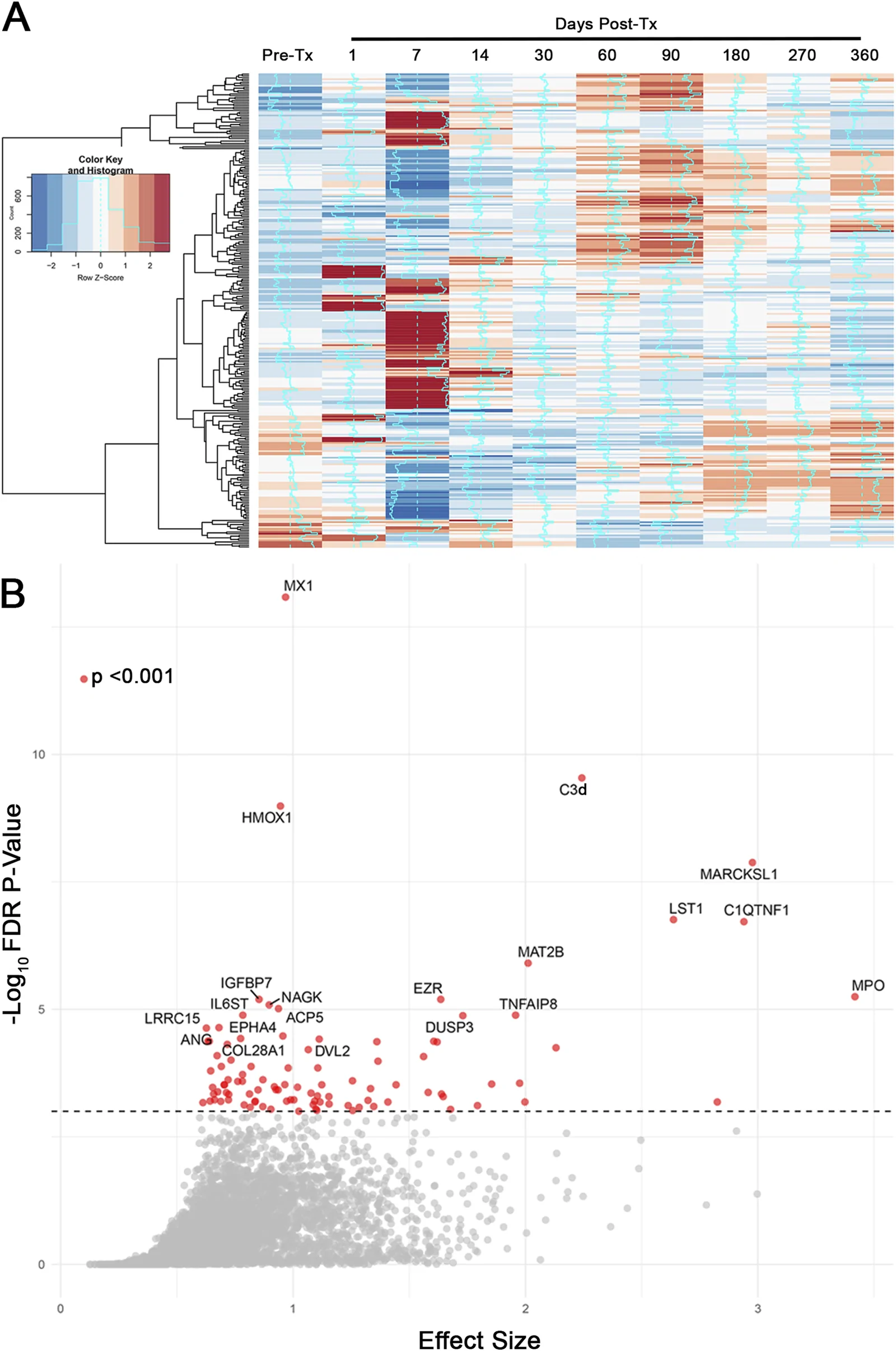
Summary of SomaScan proteomic analysis of plasma from rAAV-treated DMD boys. Plasma samples procured from that phase 1/2a trial for delandistrogene moxeparvovec in DMD boys were assayed using the SomaScan proteomics platform to identify circulating proteins responsive to intravenous treatment with high dose rAAV, relative to baseline (Pre-Tx) levels. **(A)** Heatmap of 287 significantly changed plasma protein targets (FDR p-value <0.01) across a 1-year follow-up period (n = 4 for Pre-Tx to Day 270; n = 2 for Day 360) displayed as row Z-scores relative Pre-Tx values **(B)** Volcano plot of >5,000 plasma proteins measured by SomaScan aptamer-based proteomics, with red dots indicative of 99 targets meeting a higher scrutiny threshold (FDR p-value <0.001). Data were analyzed using repeated measures ANOVA for Pre-Tx to Day 270 (α = 0.05). Dashed line **(B)** indicates FDR p-value <0.001 threshold.

**TABLE 1:** Pathway enrichment analyses associated with rAAV treatment in DMD boys.

Reactome enrichment analysis pathways	FDR P-value
Iron uptake and transport (R-hsa-917937)	1.91E-03
Collagen biosynthesis and modifying enzymes (R-hsa-1650814)	3.89E-02
Interleukin-6 signaling (R-hsa-1059683)	3.89E-02
Signaling by interleukins (R-hsa-449147)	3.89E-02
Collagen chain trimerization (R-hsa-8948216)	3.89E-02
Scavenging by class A receptors (R-hsa-3000480)	4.14E-02
Cytokine signaling in immune system (R-hsa-1280215)	4.14E-02
Senescence-associated secretory phenotype (SASP) (R-hsa-2559582)	4.14E-02
Adherens junctions interactions (R-hsa-418990)	5.23E-02
Regulation of HMOX1 expression and activity (R-hsa-9707587)	5.23E-02
KEGG pathway analysis	​
Ferroptosis (hsa04216)	1.33E-03
Complement and coagulation cascades (hsa04610)	1.33E-03
Pertussis (hsa05133)	1.33E-03
*Staphylococcus aureus* infection (hsa05150)	1.47E-03
Coronavirus disease-COVID-19 (hsa05171)	1.47E-03
Systemic lupus erythematosus (hsa05322)	6.24E-03
ECM-receptor interaction (hsa04512)	1.00E-02
Porphyrin metabolism (hsa00860)	1.32E-02
Axon guidance (hsa04360)	1.32E-02
Chagas disease (hsa05142)	1.32E-02
Gene ontogeny (GO) enrichment analysis pathways	​
Positive regulation of multicellular organismal process (GO:0051240)	1.30E-05
Positive regulation of response to stimulus (GO:0048584)	1.45E-05
System development (GO:0048731)	1.66E-05
Regulation of multicellular organismal process (GO:0051239)	2.25E-05
Positive regulation of signal transduction (GO:0009967)	2.41E-05
Anatomical structure morphogenesis (GO:0009653)	2.73E-05
Positive regulation of developmental process (GO:0051094)	3.32E-05
Regulation of response to stimulus (GO:0048583)	3.54E-05
Multicellular organism development (GO:0007275)	3.73E-05
Anatomical structure development (GO:0048856)	6.10E-05

Pathway enrichment analysis was performed on the 99 targets significantly changed relative to pre-treatment baseline (FDR p < 0.001), to determine which biological pathways are statistically overrepresented among proteins identified *via* SomaScan profiling. Analyses performed include Reactome, Gene Ontology (GO), and Kyoto Encyclopedia of Genes and Genomes (KEGG). Pathway-level FDR-adjusted p-values are shown (*FDR p < 0.05).

Following this broad analysis of SomaScan data, we investigated target dynamics across the time course of this study, relative to Pre-Tx baseline values. Consistent with the overview heatmap of Figure 3A, volcano plots depicting fold-change from Pre-Tx baseline values indicate that substantial target changes occur in both directions at Day 7 (Supplementary Figure S2). Surprisingly, Day 90 also exhibits an appreciable number of significantly increased targets, indicating delayed systemic responses to either rAAV treatment or discontinuation of daily prednisolone at Day 30 (Supplementary Figures S3-S4). As mentioned above, some of the most highly enriched pathways predicted by this dataset include those associated with immune activation and infection responses. In agreement, we find robust activation of interferon (IFN) response targets, including MX1, IFIT3, IFI16, and TLR5, that peak at Day 7 and largely return to baseline by Day 14 (Figure 4A). Activation of the complement system, which has been associated with SAEs (Lek et al., 2023a; Byrne et al., 2025a; Byrne et al., 2025b; Maurizi et al., 2026), also follows this trend, with significant elevations in C3d, C1QTNF1, and C1S peak at Day 7, whereas C1QA/C1QB/C1QC, which are consumed during complement activation (van de Bovenkamp et al., 2021), are significantly decreased at Day 14 (Figure 4B). Markers of myeloid cells and macrophages, including HMOX1, LST1, and MARCKSL1 also peak in abundance at Day 7, while IL1R1 shows significant elevations at Day 60 and Day 90 (Figure 4C). Furthermore, neutrophil markers, including MPO, LCP1, EZR, and FTH1/FTL, also agree with this sharp activation of immune responses at Day 7 (Figure 4D). These findings indicate that robust immune activation occurs following rAAV treatment in DMD boys, even in the absence of SAEs.

**FIGURE 4:**
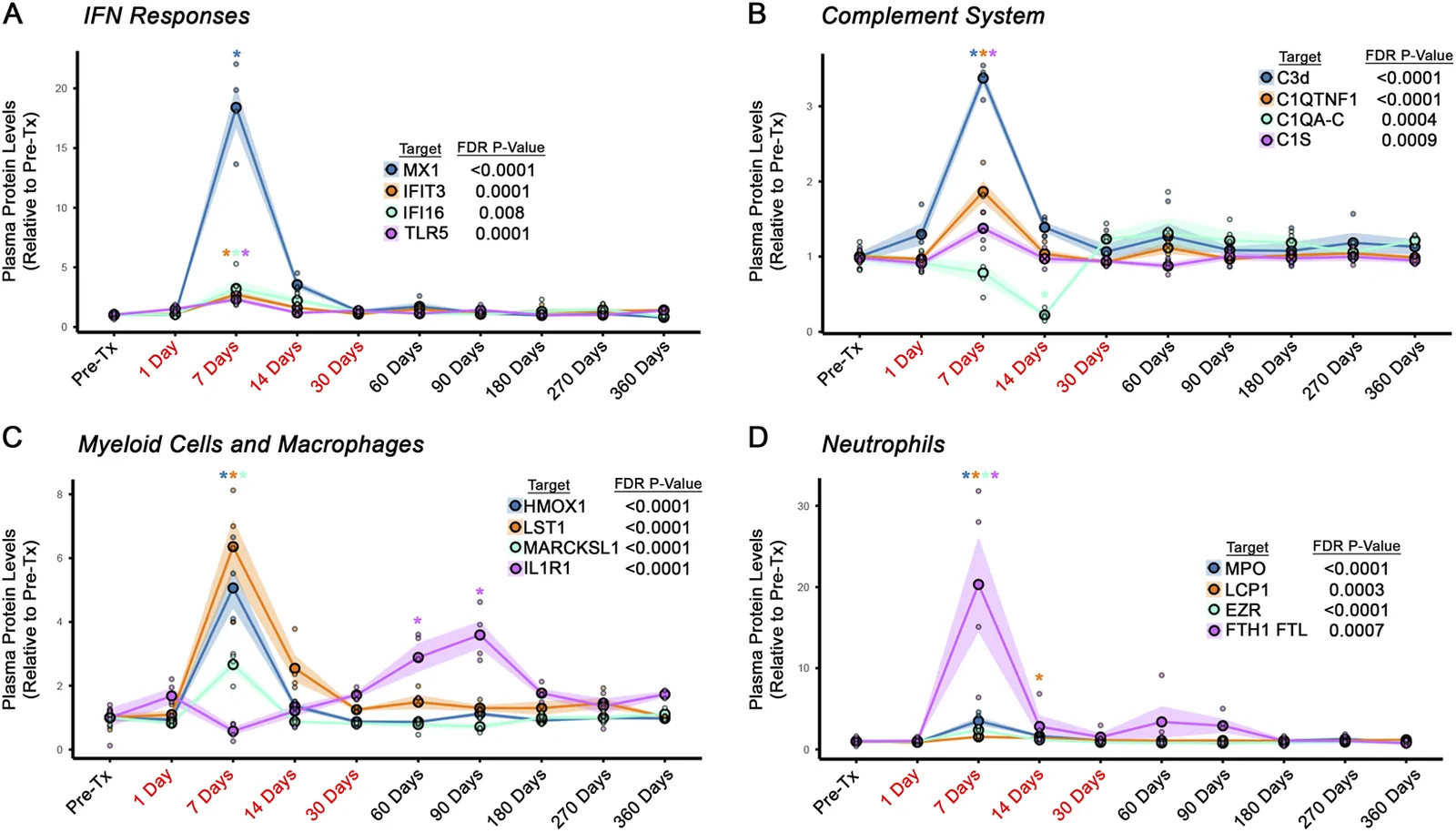
Immune-associated markers identified in rAAV-treated DMD plasma. Circulating markers associated with immune activation by delandistrogene moxeparvovec treatment in young DMD boys are displayed, including proteins associated with **(A)** interferon responses, **(B)** complement system, **(C)** myeloid cells and macrophages, and **(D)** neutrophils, as measured by SomaScan aptamer-based proteomic profiling (n = 4 for Pre-Tx to Day 270; n = 2 for Day 360). Plasma proteins are expressed relative to baseline (Pre-Tx) values (mean normalized to 1.0) and were analyzed using repeated measures ANOVA (α = 0.05; Tukey *post hoc* tests; *p < 0.05 vs. color-matched Pre-Tx values). Large open circles connected by a line represent color-matched target protein means, as indicated in legend, and small circles represent individual values. Shaded-regions represent the standard error of the mean (SEM) for respective color-matched targets. Timepoints indicated by red font indicate interval of daily steroid regimen.

The primary objective of micro-dystrophin gene therapy is to improve sarcolemmal stability to reduce muscle degeneration and slow disease progression. Therefore, we investigated muscle-associated targets within this dataset. While no obvious muscle proteins were found among the most significantly changed targets (Figure 3, Supplementary Figures S2-S4; Supplementary Table S2), significant decreases were sustained for the contractility-associated proteins TNNI3 (cardiac troponin I) and MYL6B (type I myofiber-associated myosin light chain) across the study (Figure 5A). This indicates some level of protection of cardiac and slow muscle fibers. Interestingly, plasma levels of TTN (titin) spike at Day 1 and return to near baseline values afterwards (Figure 5A), suggesting transient striated muscle damage occurs soon after treatment administration. The myotendinous junction-associated protein, TNMD (tenomodulin) is also responsive to treatment, peaking at Day 7 (Figure 5A). Other proteins that are highly expressed in muscle and affected by treatment include UBE2S, which shows a sustained decrease in plasma levels, and CA3, ACYP2, and FSTL1, which exhibit significant repeated measure effects without significance from baseline at any specific time point (Figure 5B). Surprisingly, the key skeletal and cardiac muscle damage markers of TNNI2 (fast troponin I), TNNT2 (cardiac troponin T), CKB/CKM (creatine kinase), and MYL3 (cardiac myosin light chain) were unaffected by micro-dystrophin gene therapy in these individuals (Figure 5C). These findings indicate that some level of sustained cardiac and slow skeletal muscle protection can be detected following micro-dystrophin gene therapy in DMD boys, whereas plasma levels of general and fast myofiber associated proteins are less conclusive as evidence of treatment efficacy.

**FIGURE 5:**
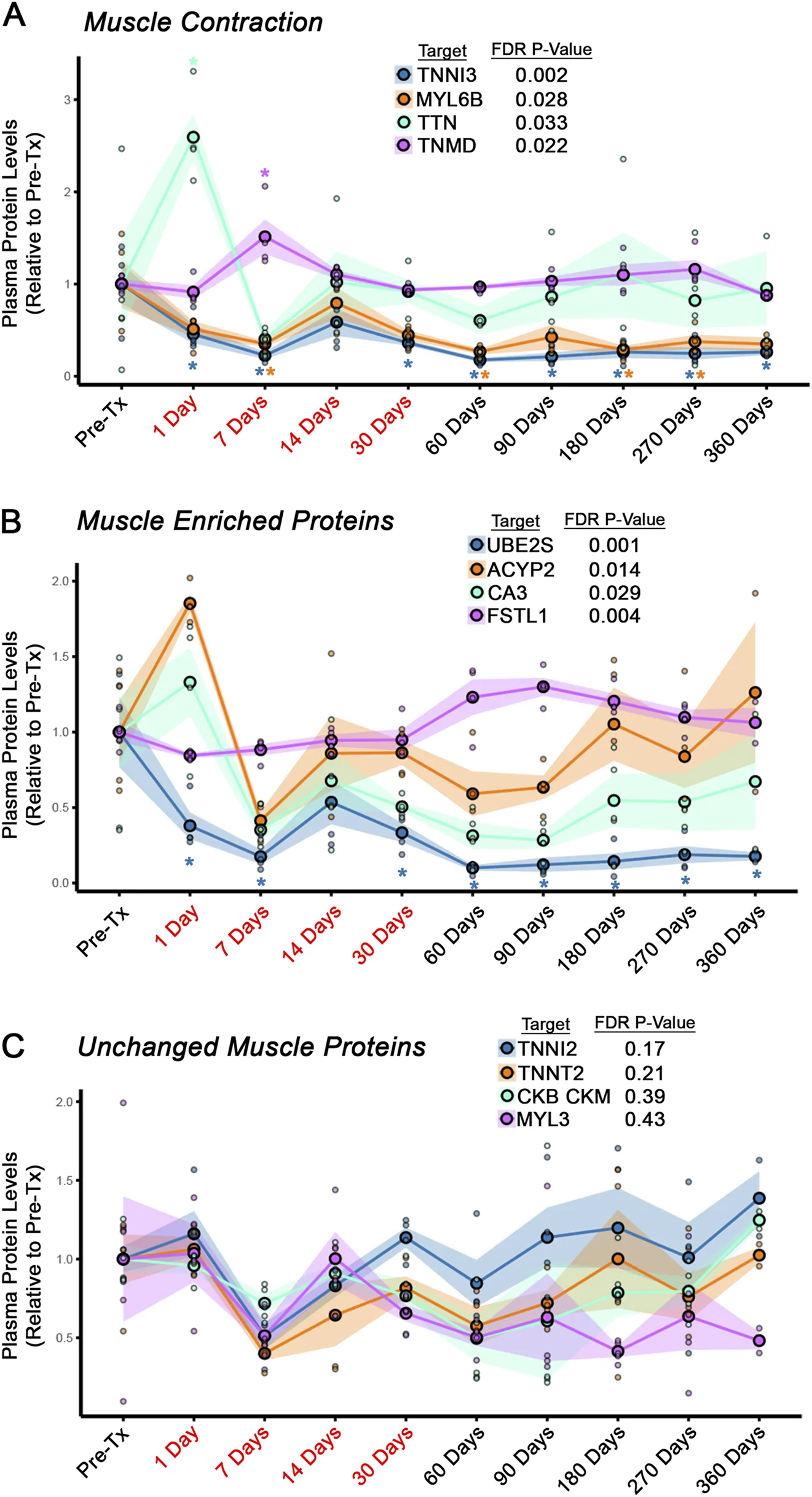
Muscle-associated markers identified in rAAV-treated DMD plasma. Circulating muscle-associated markers affected by delandistrogene moxeparvovec treatment in young DMD boys are displayed, including those classified as **(A)** muscle contraction proteins, **(B)** muscle enriched proteins, and **(C)** unchanged muscle proteins, as measured by SomaScan aptamer-based proteomic profiling (n = 4 for Pre-Tx to Day 270; n = 2 for Day 360). Plasma proteins are expressed relative to baseline (Pre-Tx) values (mean normalized to 1.0) and were analyzed using repeated measures ANOVA (α = 0.05; Tukey *post hoc* tests; *p < 0.05 vs. color-matched Pre-Tx values). Large open circles connected by a line represent color-matched target protein means, as indicated in legend, and small circles represent individual values. Shaded-regions represent the standard error of the mean (SEM) for respective color-matched targets. Timepoints indicated by red font indicate interval of daily steroid regimen.

In addition to protein targets associated with immune activation and muscle protection, we queried this SomaScan dataset for other pathways/responses of interest. As ECM associated processes were identified in Reactome and KEGG pathways analyses (Table 1), several matricellular targets showed significant dynamics following rAAV treatment, including COL28A1, COL6A3, COL1A1, and FAP (Figure 6A). In general, these proteins showed significant declines through Day 30, followed by increases to baseline levels, suggestive of effects mediated by daily prednisolone instead of rAAV treatment. Because pathways associated with axon guidance and morphogenesis were also noted in the pathway analyses, we identified interesting trends in the neuron-associated markers of RBM28 (peaks at Day 1), SHANK3 (peaks at Day 7), and ALCAM (peaks at Day 90) that suggest micro-dystrophin gene therapy may impact some neuronal/neuromuscular processes (Figure 6B). Due to some instances of liver toxicity and failure in rAAV-treated individuals (Lek et al., 2023a; Maurizi et al., 2026), we also investigated organ injury markers amongst these data. Significant hits were confirmed for HAVCR1 [also known kidney injury molecule (KIM)-1], the liver injury marker FBP1, and the broad cell damage marker IGFBP7 (Figure 7A), which all exhibited significant peaks from baseline abundance at Day 60. In agreement with the occurrence of organ injury as a result of rAAV gene therapy, levels of growth and differentiation factor (GDF) 15, an acute injury response protein (Baek and Eling, 2019), demonstrated elevations from Day 7 to Day 90 (Figure 7B). Other GDF ligands, including GDF11/MSTN, GDF9, and GDF5, also showed significant effects across the time course, with GDF9 and GDF5 showing significant peaks at Day 7, relative to Pre-Tx levels (Figure 7B). Additional targets associated with the TGFβ superfamily, including TGFB1, TGFB2, and TGFBR3, also exhibited significance in this dataset, with the TGFB1 and TGFB2 ligands also peaking at Day 7 (Figure 7C). Individual participant trajectories for MX1, ILR1R1, TNNI3, and GDF15 are shown Supplementary Figure S5. These findings illustrate the complexity of systemic responses that occur in individuals following intravenous rAAV administration and may provide useful insights for participant monitoring and/or identification of interventional mechanisms to improve the safety of rAAV in the clinic. It is important to consider with the interpretation of these results that samples from age-matched healthy, untreated, or glucocorticoid regimen DMD controls were not available for analysis in this study.

**FIGURE 6:**
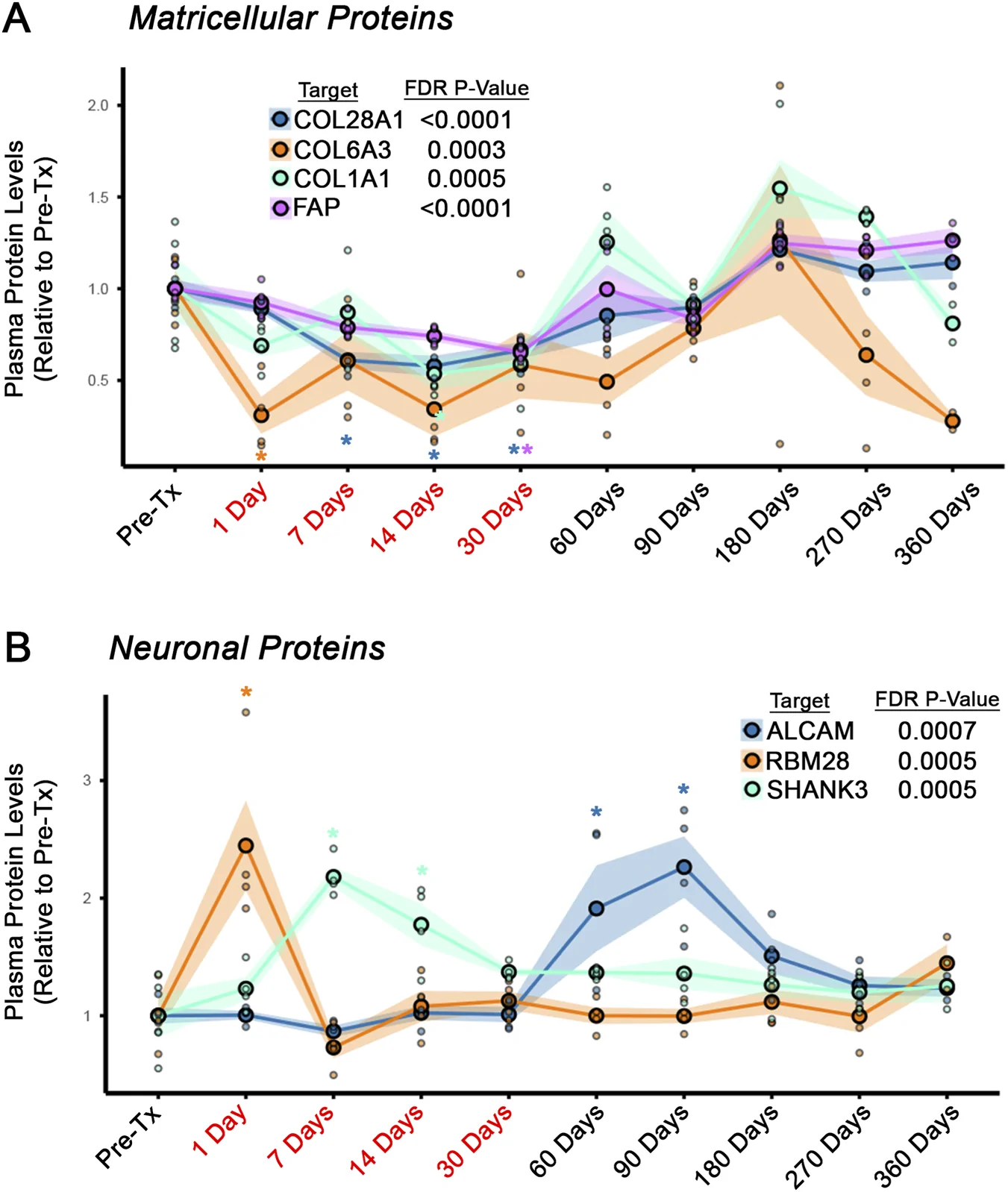
Matricellular- and neuronal-associated markers identified in rAAV-treated DMD plasma. Circulating markers affected by delandistrogene moxeparvovec treatment in young DMD boys are displayed, including those classified as **(A)** matricellular proteins and **(B)** neuronal proteins, as measured by SomaScan aptamer-based proteomic profiling (n = 4 for Pre-Tx to Day 270; n = 2 for Day 360). Plasma proteins are expressed relative to baseline (Pre-Tx) values (mean normalized to 1.0) and were analyzed using repeated measures ANOVA (α = 0.05; Tukey *post hoc* tests; *p < 0.05 vs. color-matched Pre-Tx values). Large open circles connected by a line represent color-matched target protein means, as indicated in legend, and small circles represent individual values. Shaded-regions represent the standard error of the mean (SEM) for respective color-matched targets. Timepoints indicated by red font indicate interval of daily steroid regimen.

**FIGURE 7:**
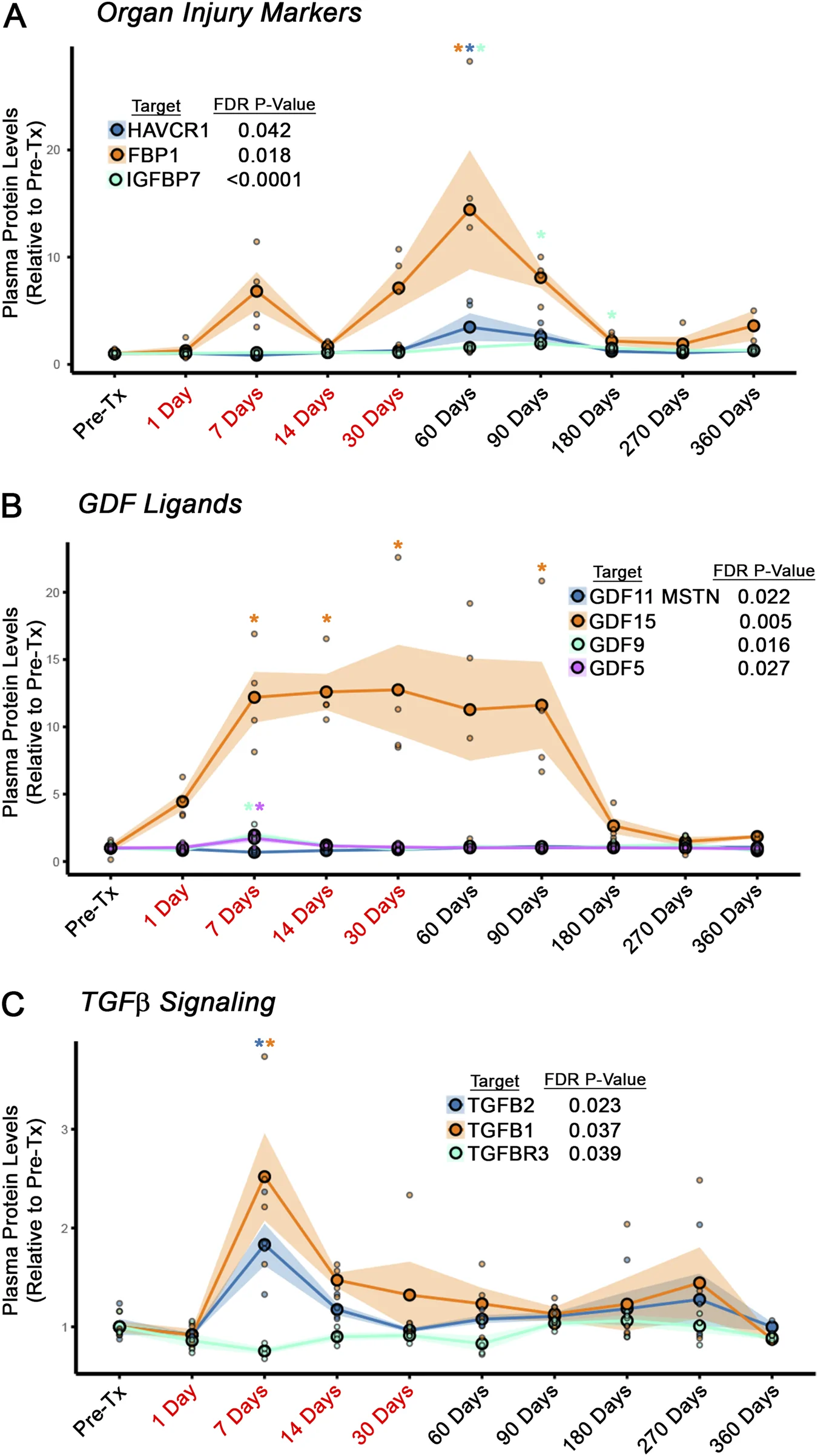
Markers of organ injury and pathological processes identified in rAAV-treated DMD plasma. Circulating markers affected by delandistrogene moxeparvovec treatment in young DMD boys are displayed, including those associated with **(A)** organ injury, **(B)** GDF ligands, and **(C)** TGFβ signaling, as measured by SomaScan aptamer-based proteomic profiling (n = 4 for Pre-Tx to Day 270; n = 2 for Day 360). Plasma proteins are expressed relative to baseline (Pre-Tx) values (mean normalized to 1.0) and were analyzed using repeated measures ANOVA (α = 0.05; Tukey *post hoc* tests; *p < 0.05 vs. color-matched Pre-Tx values). Large open circles connected by a line represent color-matched target protein means, as indicated in legend, and small circles represent individual values. Shaded-regions represent the standard error of the mean (SEM) for respective color-matched targets. Timepoints indicated by red font indicate interval of daily steroid regimen.

### Gene expression responses to rAAV in livers of dystrophic mice

Following analysis of SomaScan data, we sought to determine if aspects of the human systemic response to rAAV administration, particularly antiviral and IFN-associated responses, are exhibited in preclinical models of DMD. To investigate this prospect, 6 month-old male mice of the severe D2. *mdx* mouse model of DMD (Hammers et al., 2020a) received systemic control or rAAV treatment (IV; rAAV dose of 2 × 10^14^ vg/kg). One week following treatment, livers were harvested and analyzed for gene expression of *Mx1*, *Ifit3*, *Ifnb*, and *Mavs*. While *Mx1*, which was the top overall changed protein in post-AAV DMD plasma (Supplementary Table S2), trended towards an increase in rAAV-treated mouse livers, the viral response genes *Ifit3* and *Ifnb*, as well as the viral sensing gene *Mavs*, were significantly increased by rAAV treatment (Figure 8). These data indicate that aspects of immune activation and antiviral/IFN responses to systemic rAAV treatment identified in humans do occur in dystrophic mice, offering possible outcomes to investigate mechanisms underpinning these rAAV responses and potential interventions to mitigate their occurrence. It remains to be determined how age/disease severity affects the magnitude of these responses to rAAV administration, as well as specific cell populations responsible for these observed responses.

**FIGURE 8:**
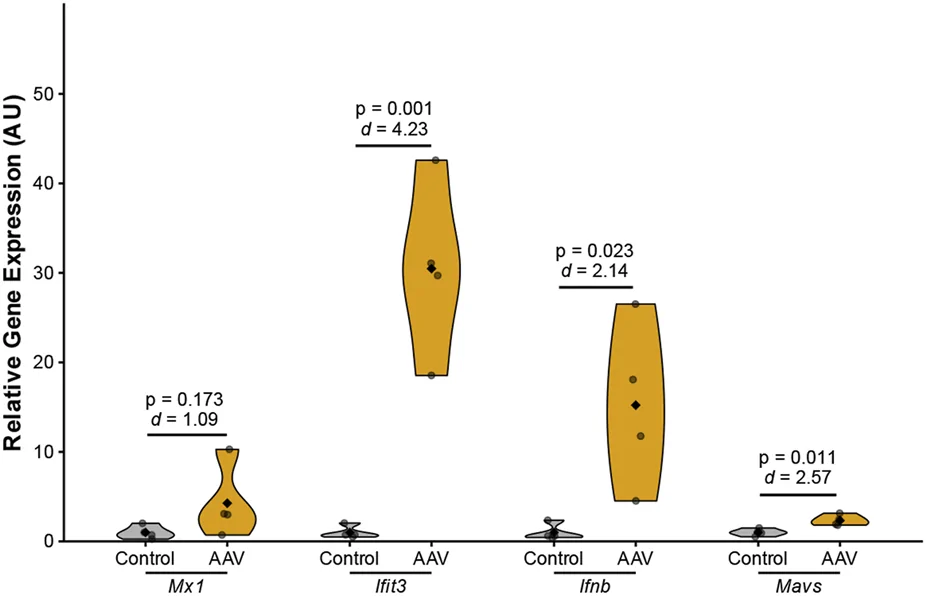
Antiviral gene expression responses to rAAV treatment in D2. *mdx* mouse livers. Male D2. *mdx* mice were treated with control or AAVrh10. EGFP (n = 4; rAAV dose of 2 × 10^14^ vg/kg) and investigated for interferon- and antiviral-associated gene expression in the liver 7 days following injection. Data are displayed as violin plots with individual values indicated by circles and means indicated by black diamonds (n = 4) and were analyzed using two-tailed unpaired Student’s t-tests (α = 0.05; p-values and effect sizes are indicated).

## Discussion

After decades of development, gene therapies utilizing intravenous administration of high-dose rAAVs are in the clinic for the treatment of human disease. While the potential transformative impact of these therapies on disease outcomes has led to optimism and momentum in the field (Mendell et al., 2024; Mendell et al., 2024; Mendell et al., 2026), reports of life-threatening adverse reactions and questionable efficacy (Lek et al., 2023a; Lek et al., 2023b; Potter et al., 2025; Maurizi et al., 2026) highlight the need to better understand rAAV responses in the clinic for both the effective and ineffective/adverse cases. In this report, we investigated the rAAV dynamics and systemic responses in biospecimens procured during the phase 1/2a trial for delandistrogene moxeparvovec (rAAVrh74. MHCK7. micro-dystrophin) in young (>4 to <8 year-old) DMD boys to gain an understanding of how these aspects are impacted in context of effective and SAE-free rAAV gene therapy. In summary, we identified that biologically active and packaged vector genomes are circulating in the plasma at high levels at Day 1 and are largely reduced by Day 7, with minimal urinary shedding. Furthermore, SomaScan proteomic analysis of plasma samples revealed robust immune responses provoked by rAAV treatment with variable impact on long-term reduction of muscle proteins in circulation. Together these findings provide new insights into the underlying biological processes that occur in humans following intravenous rAAV administrations during a “best-case scenario” that can be used to facilitate the development of improved gene therapy safety, efficacy, and monitoring.

Importantly, contextualizing these findings alongside reports of SAEs further highlights the need to define the full spectrum of systemic responses to high dose rAAV gene therapy treatment. While SAEs such as hepatotoxicity, thrombotic microangiopathy, and complement-mediated inflammation have been documented in a subset of participants (Maurizi et al., 2026), the mechanisms that differentiate transient responses from those that progress to pathology remain poorly defined. This limits the ability to predict risk, refine dosing, and intervene effectively. By detailing systemic responses in rAAV treated participants who did not experience SAEs, studies such as ours provide a critical reference point for comparison, enabling the identification of response trajectories that may precede adverse outcomes. Such insight will be central to improving clinical monitoring strategies and the overall safety profile of rAAV gene therapies.

Because rAAV can exist in a complex mix of states, including fully packaged, partially packaged and unpackaged/empty capsids, the packaging status of circulating rAAV particles has direct implications for immune responses. For instance, empty capsids have been shown to more strongly induce innate immune activation compared to full capsids (Jarvi et al., 2024). This is particularly concerning because traditional genome-based rAAV titering and dosing does not account for empty capsids (i.e., no vector genome), therefore, a batch with a higher proportion of empty capsids requires more total viral particles to be administered in order to reach the same target genome-based dose (Tereshko et al., 2023). This increases the immunogenic viral burden being delivered, further raising the risk of serious immune responses (Herzog et al., 2002).

An emerging concept to consider with systemic rAAV gene therapies is the incidence of AAV genome ejection, as recent evidence suggests that AAV2 and AAV5 can undergo spontaneous genome ejection in a temperature-dependent manner, starting at 37 °C (Gliwa et al., 2025). This raises the possibility that a portion of delivered rAAV may release its genetic material, resulting in **a**) free floating single-stranded DNA and **b**) a decrease in the effective capsid filling ratio in circulation. In our investigation of post-infusion vector content in biobanked plasma and urine specimens, we identified high vector content, as determined by both dPCR and immunoblotting, in Day 1 plasma samples and significant clearance by Day 7. Importantly, we confirmed that the majority of vector genomes detected in these samples were packaged, and vector in Day 1 plasma samples retains high transduction competency. These findings suggest that spontaneous genome ejection is not a prominent feature in the early timepoints of systemic AAVrh74 gene therapies; however, this phenomenon should be considered and investigated when different rAAV capsids are implemented in systemic gene therapies.

An important finding of the current report is the robust activation of innate immune responses following high-dose AAVrh74 administration, as identified in the SomaScan proteomic profiling of plasma samples. The innate immune response is the body’s first line of defense against viral infections, including from rAAV gene therapy. When the rAAV is administered systemically, it is sensed by pattern recognition receptors, including Toll-like receptors (TLRs), on multiple cell types (Akira et al., 2006; Xicluna et al., 2025). This triggers the type I interferon cascade, leading to IFN production and the downstream induction of interferon-stimulated genes (ISGs), such as MX1, IFIT3, and IFI16 (Chikhalya et al., 2021; Xicluna et al., 2025). Consistent with these pathways being activated in the clinical setting, our data reveal that the ISGs MX1, IFIT3, and IFI16 are highly elevated at Day 7, concurrent with vector clearance, and returns to Pre-Tx levels by Day 14. MX1 (myxovirus resistance protein 1) is an interferon-induced antiviral GTPase that blocks viral replication and is an established biomarker of type I IFN signaling and IFN-responsive cells (Haller and Kochs, 2011; Huijser et al., 2019). Among the ISGs assessed, MX1 showed the largest and most significant change in the SomaScan dataset, consistent with innate immune activation.

Given the importance of IFN-mediated pathways in antiviral responses (Schoggins and Rice, 2011) and their potential involvement in rAAV-related SAE development, we investigated involvement of these pathways in preclinical rAAV responses in a severe mouse model of DMD. Consistent with clinical plasma protein levels, rAAV treatment significantly elevated *Ifit3* gene expression in mouse livers 7 days following treatment, suggesting IFIT3 is a translational biomarker of rAAV systemic response that could be potentially leveraged to assess the magnitude of antiviral responses to rAAV. Additionally, the IFN-associated antiviral response genes *Ifnb* and *Mavs* were also elevated in response to clinical rAAV systemic doses. Interestingly, our most prominent hit in plasma, MX1, only trended towards an increase in rAAV-treated livers. This is likely attributed to murine *Mx1* mutations in most inbred mouse genetic backgrounds (Staeheli et al., 1988) and suggests that MX1 may not be an effective biomarker for comparing murine and human responses to rAAV. MX1 responses in other preclinical DMD models, such as golden retriever muscular dystrophy canines and DMD rats (Hammers et al., 2016; Lee et al., 2026), should be investigated to determine its utility as a translational biomarker for rAAV responses. Additionally, capsid- and vector genome-specific responses should be investigated to determine if differential responses occur with different capsids and/or transgenes. These data, nonetheless, serve as a strong proof-of-concept for future testing.

Markers associated with macrophages and neutrophils were also prominently activated following rAAV treatment, with both macrophage (HMOX1, LST1, MARCKSL1) and neutrophil markers (MPO, LCP1, EZR, FTH1 FTL) peaking at Day 7 and resolving by Day 14. One exception is IL1R1, an interleukin-1 receptor, that signals through the IL-1 pathway. Unlike the macrophage and neutrophil markers, IL1R1 showed a delayed response to rAAV exposure, peaking at Day 60 to Day 90, well beyond the vector clearance window. Notably, this elevation coincides with the steroid taper period. As glucocorticoids are known to suppress IL-1β gene transcription and mRNA stability, the observed rise may reflect rebound IL-1 pathway activity following a reduction in glucocorticoid-mediated immune suppression rather than a direct response to rAAV exposure (Knudsen et al., 1987; Lee et al., 1988). Across the IFN, macrophage, and neutrophil panels, we observe evidence of an immune response consistent with an acute, self-limiting innate response to rAAV vector exposure. This is evidenced by the temporal alignment of the observed immune response to vector kinetics, with a spike in classical innate immune markers at Day 7 as vector is cleared and a return to baseline by Day 14. This acute, self-limiting pattern seen in boys without SAEs contrasts with the sustained immune response seen in participants with SAEs (Emami et al., 2024; Leon-Astudillo et al., 2026).

The complement system has been identified as a major component of rAAV responses and possibly contributes to SAEs following rAAV gene therapy (Lek et al., 2023b; Salabarria et al., 2024; Byrne et al., 2025a). Consistently, we identify markers of complement activation were also increased in plasma samples from gene therapy-treated DMD boys who experienced only mild-moderate side effects. This suggests that complement activation is a natural, and likely ubiquitous response to rAAV therapies. These findings, therefore, reinforce the need to further develop strategies for preventing, monitoring, and controlling the complement cascade in gene therapy participants to help facilitate the safe administration of rAAV-based therapeutics.

Limiting immune responses in individuals treated with rAAV has important implications in reducing SAEs and increasing safety. For example, the anti-capsid antibodies produced following rAAV therapy can impact the participant’s eligibility for AAV re-administration (Rana et al., 2024)**.** Additionally, participants with pre-existing anti-AAV antibodies are ineligible for this type of treatment (Saha et al., 2026)**.** Recent studies addressing these concerns have shown that immunosuppression strategies, including transient B-cell depletion (Rana et al., 2024) and plasma cell-directed therapy (Saha et al., 2026)**,** may be able to address these concerns in rAAV treatment to enable rAAV re-administration or administration in participants with pre-existing immunity.

The primary goal of micro-dystrophin gene therapy is to express a miniaturized dystrophin transgene to provide functional sarcolemmal stability and reduce mechanical injury to muscle cells. Therefore, circulating biomarkers of muscle damage, which typically include muscle-specific energetic, structural, and contractile proteins that are released into circulation through disrupted myofiber membranes (Allen and Whitehead, 2011), should serve as reliable indicators of treatment efficacy by providing a readout of skeletal and cardiac muscle injury and disease progression (Hathout et al., 2015). In this report, we identify TNNI3 and MYL6B as striated muscle injury biomarkers that exhibit sustained systemic reduction following micro-dystrophin gene therapy. While their treatment-associated decreases are in agreement with sustained functional improvements achieved by delandistrogene moxeparvovec treatment in these individuals (Mendell et al., 2024; Mendell et al., 2026), it is interesting that these markers are expressed primarily in the heart (TNNI3) and slow-twitch muscle (MYL6B). On the contrary, logical circulating biomarkers of skeletal muscle correction, including CKB/CKM and TNNI2, were shown to be unchanged by micro-dystrophin treatment *via* SomaScan assays. Intriguingly, circulating TTN levels elevate substantially at Day 1, suggesting some level of infusion-associated muscle damage is occurring.

Other muscle enriched proteins (ACYP2, CA3, FSTL1), showed a significant repeated-measures effect without individual timepoint significance. CA3 has been reported as a sensitive damage marker in participants with DMD, with circulating levels reported 35- to 51-fold higher in untreated DMD participants compared to healthy controls (Johansson et al., 2023; Ahmadiharchegani et al., 2025). FSTL1 is a multifunctional glycoprotein that is associated with tissue repair in both skeletal and cardiac muscle (He et al., 2025). In our dataset, CA3 trended downwards and FSTL1 trended upwards across the time course. This pattern may be consistent with a reduced muscle turnover and increased regenerative response following treatment. However, declines in muscle-derived circulating proteins, including CA3, have been reported with disease progression alone in DMD cohorts (Hathout et al., 2015). Interestingly, UBE2S, an E2 ubiquitin-conjugating enzyme involved in proteasome-mediated protein degradation (Garnett et al., 2009), showed a sustained decrease across the time course. While UBE2S’s specific role in striated muscle is currently unclear, UBE2-class proteins are known to have roles in muscle protein quality control and atrophy (Polge et al., 2015). UBE2S has been shown to work in tandem with UBE2C to ubiquitinate APC/C structures for degradation in cell cycle regulation (Martinez-Chacin et al., 2020), and UBE2C, on its own, has been found to promote skeletal muscle regeneration and myoblast differentiation (Yuan et al., 2024). Systemic decreases in UBE2S, therefore, could reflect a decrease in this pathway regulating muscle regeneration and differentiation, suggesting lower levels of muscle turnover is occurring in these young boys. These aspects remain to be mechanistically explored, however, our findings suggest UBE2S may be a novel circulating biomarker of micro-dystrophin efficacy.

Interestingly, we see early suppression of matricellular proteins, including COL28A1, COL6A3, COL1A1, and FAP, through Day 30, coinciding with the daily glucocorticoid regimen. Corticosteroids are known to influence ECM remodeling through modulation of collagen and ECM protein synthesis (Cutroneo and Sterling, 2004; Jarvelainen et al., 2009), therefore, the early suppression and subsequent rebound of matricellular proteins observed here is likely attributable to changes in glucocorticoid regimens rather than rAAV exposure and/or impact of the micro-dystrophin transgene.

The neuron-associated proteins SHANK3, RBM28, and ALCAM also showed significant effects in this study. SHANK3, a post-synaptic scaffolding protein that is classically linked to neurodevelopmental disorders including autism spectrum disorder (Durand et al., 2007), is elevated at Day 7 and Day 14. In the context of striated muscle, SHANK3 is localized in the Z-discs of sarcomeres and serves as a marker of neuromuscular junction integrity [NMJ; (Lutz et al., 2020)]. Elevation of SHANK3 could suggest an increase in the stabilization of the NMJ as transgene is expressed, however, it is important to note that its upregulation is transient, returning to baseline by Day 30, suggesting potential involvement in early muscle remodeling caused by micro-dystrophin and/or glucocorticoids. RBM28, a nucleolar RNA-binding protein involved in ribosomal RNA processing and ribosome biogenesis (Damianov et al., 2006), showed a dramatic spike at Day 1. This protein has been implicated in the nucleolar stress response (Lin et al., 2022), and transient elevation may reflect a nuclear stress response due to acute cellular stress associated with vector infusion (Boulon et al., 2010), potentially involving viral infection or upregulation of protein synthesis machinery in response to transgene delivery. ALCAM, a cell adhesion molecule involved in neural development and immune cell migration (Swart, 2002), showed a delayed elevation peaking at Day 60 to Day 90, the significance of which in this context remains unclear. Notably, ALCAM levels peak following the corticosteroid taper period, which may reflect immune reactivation as glucocorticoid-mediated suppression is reduced.

Another important finding in this report is the delayed emergence of the organ injury markers HAVCR1, FBP1, and IGFBP7 that peak at Day 60 to Day 90. While it is currently unclear if these systemic increases are associated with the rAAV treatment, glucocorticoid reduction, or a combination of the two effects, these markers may serve as useful indicators of unintended organ damage during rAAV treatment protocols. Related to organ stress/injury, the early and persistent elevation of GDF15, an early response tissue stress signaling protein (Baek and Eling, 2019), is also intriguing, as it appears to not be associated with altered glucocorticoid regimen. While other GDFs and TGFβ primarily peak with other immune markers at Day 7, GDF15 maintains high levels for at least 3 months. While this is, to our knowledge, the first direct association of increased GDF15 plasma levels to rAAV gene therapy, there has been an association of fetal-derived GDF15 to the nausea and vomiting during pregnancy (Fejzo et al., 2024), and circulating GDF15 is linked to high-emesis scores during chemotherapy treatments (Breen et al., 2020). Given that all participants in this clinical trial experienced vomiting (Mendell et al., 2020) and vomiting is one of the most prevalent AEs associated with systemic rAAV gene therapies (Duan, 2023), sustained post-infusion elevations in circulating GDF15 provide a possible explanation for this common treatment-associated AE. Therefore, a potential therapeutic intervention against rAAV-induced vomiting may be treatment with anti-GDF15 antibodies, which are currently in the clinic for the treatment of cancer cachexia (Groarke et al., 2024).

This study does have several limitations. For instance, the inclusion of healthy and untreated DMD controls in the SomaScan analysis would enable better insight into how proteins modified by delandistrogene moxeparvovec treatment, such as TNNI3, MYL6B, and UBE2S, compare with natural trajectories in age-matched healthy and DMD boys. Similarly, the inclusion of age-matched controls for the glucocorticoid regimen would allow us to discern rAAV- from drug-induced effects, as noted above for the matricellular protein panel. Furthermore, additional sampling during the early post-treatment period (i.e., between Day 1 and Day 14) would provide better granularity of both rAAV dynamics and the activation and peaks of the immune and inflammatory markers that dominate the early rAAV response. This study, importantly, will inform the scientific and clinical communities of these considerations, which should aid in the design of future studies looking to investigate these features, as well as provide insights into potential immune and inflammatory pathways that can be targeted to improve the safety of systemic rAAV gene therapies.

## Data Availability

The original contributions presented in the study are included in the article/supplementary material, further inquiries can be directed to the corresponding author.

## References

[B1] Ahmadiharchegani F. Tobin R. A. Degan C. Naveed A. Guglieri M. Jimenez-Requena A. (2025). Circulating protein biomarkers identified in two independent clinical trial cohorts of glucocorticoid-naive Duchenne muscular dystrophy patients. Sci. Rep. 15 (1), 39997. 10.1038/s41598-025-23758-6 PMC12618936 41238663

[B2] Akira S. Uematsu S. Takeuchi O. (2006). Pathogen recognition and innate immunity. Cell 124 (4), 783–801. 10.1016/j.cell.2006.02.015 16497588

[B3] Allen D. G. Whitehead N. P. (2011). Duchenne muscular dystrophy-what causes the increased membrane permeability in skeletal muscle? Int. J. Biochem. Cell Biol. 43 (3), 290–294. 10.1016/j.biocel.2010.11.005 21084059

[B4] Baek S. J. Eling T. (2019). Growth differentiation factor 15 (GDF15): a survival protein with therapeutic potential in metabolic diseases. Pharmacol. Ther. 198, 46–58. 10.1016/j.pharmthera.2019.02.008 30790643 PMC7196666

[B5] Boulon S. Westman B. J. Hutten S. Boisvert F. M. Lamond A. I. (2010). The nucleolus under stress. Mol. Cell 40 (2), 216–227. 10.1016/j.molcel.2010.09.024 20965417 PMC2987465

[B6] Breen D. M. Kim H. Bennett D. Calle R. A. Collins S. Esquejo R. M. (2020). GDF-15 neutralization alleviates platinum-based chemotherapy-induced Emesis, anorexia, and weight loss in mice and nonhuman Primates. Cell Metab. 32 (6), 938–950 e936. 10.1016/j.cmet.2020.10.023 33207247

[B7] Byrne B. J. Butterfield R. J. Shieh P. B. Smith E. C. Licht C. Binks M. (2025a). Complement activation in a phase Ib study of fordadistrogene movaparvovec for Duchenne muscular dystrophy. Mol. Ther. 33 (9), 4226–4238. 10.1016/j.ymthe.2025.06.032 PMC12432875 40583275

[B8] Byrne B. J. Flanigan K. M. Matesanz S. E. Finkel R. S. Waldrop M. A. D'Ambrosio E. S. (2025b). Current clinical applications of AAV-Mediated gene therapy. Mol. Ther. 33, 2479–2516. 10.1016/j.ymthe.2025.04.045 PMC12172329 40329530

[B9] Chikhalya A. Dittmann M. Zheng Y. Sohn S. Y. Rice C. M. Hearing P. (2021). Human IFIT3 protein induces interferon signaling and inhibits adenovirus immediate early gene expression. mBio 12 (6), e0282921. 10.1128/mBio.02829-21 PMC8561380 34724821

[B10] Cutroneo K. R. Sterling K. M. Jr. (2004). How do glucocorticoids compare to oligo decoys as inhibitors of collagen synthesis and potential toxicity of these therapeutics? J. Cell Biochem. 92 (1), 6–15. 10.1002/jcb.20030 15095399

[B11] Damianov A. Kann M. Lane W. S. Bindereif A. (2006). Human RBM28 protein is a specific nucleolar component of the spliceosomal snRNPs. Biol. Chem. 387 (10-11), 1455–1460. 10.1515/BC.2006.182 17081119

[B12] Duan D. (2023). Lethal immunotoxicity in high-dose systemic AAV therapy. Mol. Ther. 31 (11), 3123–3126. 10.1016/j.ymthe.2023.10.015 PMC10638066 37822079

[B13] Durand C. M. Betancur C. Boeckers T. M. Bockmann J. Chaste P. Fauchereau F. (2007). Mutations in the gene encoding the synaptic scaffolding protein SHANK3 are associated with autism spectrum disorders. Nat. Genet. 39 (1), 25–27. 10.1038/ng1933 17173049 PMC2082049

[B14] Emami M. R. Brimble M. A. Espinoza A. Owens J. Whiteley L. O. Casinghino S. (2024). Single cell and TCR analysis of immune cells from AAV gene therapy-dosed Duchenne muscular dystrophy patients. Mol. Ther. Methods Clin. Dev. 32 (4), 101349. 10.1016/j.omtm.2024.101349 PMC11546459 39524974

[B15] England S. B. Nicholson L. V. Johnson M. A. Forrest S. M. Love D. R. Zubrzycka-Gaarn E. E. (1990). Very mild muscular dystrophy associated with the deletion of 46% of dystrophin. Nature 343 (6254), 180–182. 10.1038/343180a0 2404210

[B16] Fejzo M. Rocha N. Cimino I. Lockhart S. M. Petry C. J. Kay R. G. (2024). GDF15 linked to maternal risk of nausea and vomiting during pregnancy. Nature 625 (7996), 760–767. 10.1038/s41586-023-06921-9 PMC10808057 38092039

[B17] Garnett M. J. Mansfeld J. Godwin C. Matsusaka T. Wu J. Russell P. (2009). UBE2S elongates ubiquitin chains on APC/C substrates to promote mitotic exit. Nat. Cell Biol. 11 (11), 1363–1369. 10.1038/ncb1983 19820702 PMC2875106

[B18] Gliwa K. Hull J. Kansol A. Zembruski V. Lakshmanan R. Mietzsch M. (2025). Biophysical and structural insights into AAV genome ejection. J. Virol. 99 (3), e0089924. 10.1128/jvi.00899-24 PMC11915859 39907279

[B19] Gold L. Ayers D. Bertino J. Bock C. Bock A. Brody E. N. (2010). Aptamer-based multiplexed proteomic technology for biomarker discovery. PLoS One 5 (12), e15004. 10.1371/journal.pone.0015004 21165148 PMC3000457

[B20] Groarke J. D. Crawford J. Collins S. M. Lubaczewski S. Roeland E. J. Naito T. (2024). Ponsegromab for the treatment of cancer cachexia. N. Engl. J. Med. 391 (24), 2291–2303. 10.1056/NEJMoa2409515 39282907

[B21] Haller O. Kochs G. (2011). Human MxA protein: an interferon-induced dynamin-like GTPase with broad antiviral activity. J. Interferon Cytokine Res. 31 (1), 79–87. 10.1089/jir.2010.0076 21166595

[B22] Hammers D. W. Sleeper M. M. Forbes S. C. Shima A. Walter G. A. Sweeney H. L. (2016). Tadalafil treatment delays the onset of cardiomyopathy in dystrophin-deficient hearts. J. Am. Heart Assoc. 5 (8), e003911. 10.1161/JAHA.116.003911 27506543 PMC5015305

[B23] Hammers D. W. Merscham-Banda M. Hsiao J. Y. Engst S. Hartman J. J. Sweeney H. L. (2017). Supraphysiological levels of GDF11 induce striated muscle atrophy. EMBO Mol. Med. 9 (4), 531–544. 10.15252/emmm.201607231 28270449 PMC5376753

[B24] Hammers D. W. Hart C. C. Matheny M. K. Wright L. A. Armellini M. Barton E. R. (2020a). The D2.mdx mouse as a preclinical model of the skeletal muscle pathology associated with Duchenne muscular dystrophy. Sci. Rep. 10 (1), 14070. 10.1038/s41598-020-70987-y 32826942 PMC7442653

[B25] Hammers D. W. Hart C. C. Patsalos A. Matheny M. K. Wright L. A. Nagy L. (2020b). Glucocorticoids counteract hypertrophic effects of myostatin inhibition in dystrophic muscle. JCI Insight 5 (1), e133276. 10.1172/jci.insight.133276 31830002 PMC7030817

[B26] Harper S. Q. Hauser M. A. DelloRusso C. Duan D. Crawford R. W. Phelps S. F. (2002). Modular flexibility of dystrophin: implications for gene therapy of Duchenne muscular dystrophy. Nat. Med. 8 (3), 253–261. 10.1038/nm0302-253 11875496

[B27] Hathout Y. Brody E. Clemens P. R. Cripe L. DeLisle R. K. Furlong P. (2015). Large-scale serum protein biomarker discovery in Duchenne muscular dystrophy. Proc. Natl. Acad. Sci. U. S. A. 112 (23), 7153–7158. 10.1073/pnas.1507719112 26039989 PMC4466703

[B28] He F. Wang X. Man Y. Xu Q. Yi X. Liu J. (2025). Impact of follistatin-like 1 on cardiac repair processes following myocardial infarction. Front. Cell Dev. Biol. 13, 1746363. 10.3389/fcell.2025.1746363 PMC12833442 41602834

[B29] Herzog R. W. Fields P. A. Arruda V. R. Brubaker J. O. Armstrong E. McClintock D. (2002). Influence of vector dose on factor IX-specific T and B cell responses in muscle-directed gene therapy. Hum. Gene Ther. 13 (11), 1281–1291. 10.1089/104303402760128513 12162811

[B30] Hoffman E. P. Brown R. H. Jr. Kunkel L. M. (1987). Dystrophin: the protein product of the Duchenne muscular dystrophy locus. Cell 51 (6), 919–928. 10.1016/0092-8674(87)90579-4 3319190

[B31] Huijser E. van Helden-Meeuwsen C. G. Groot N. Bodewes I. L. A. Wahadat M. J. Schreurs M. W. J. (2019). MxA is a clinically applicable biomarker for type I interferon activation in systemic lupus erythematosus and systemic sclerosis. Rheumatol. Oxf. 58 (7), 1302–1303. 10.1093/rheumatology/kez078 30879072

[B32] Jarvelainen H. Sainio A. Koulu M. Wight T. N. Penttinen R. (2009). Extracellular matrix molecules: potential targets in pharmacotherapy. Pharmacol. Rev. 61 (2), 198–223. 10.1124/pr.109.001289 19549927 PMC2830117

[B33] Jarvi N. Hofman K. Venkatesh A. Gorecki E. Balu-Iyer S. V. (2024). Immunogenicity risk assessment of empty capsids present in adeno-associated viral vectors using predictive innate immune responses. J. Pharm. Sci. 113 (12), 3457–3469. 10.1016/j.xphs.2024.09.006 PMC13286125 39326842

[B34] Johansson C. Hunt H. Signorelli M. Edfors F. Hober A. Svensson A. S. (2023). Orthogonal proteomics methods warrant the development of Duchenne muscular dystrophy biomarkers. Clin. Proteomics 20 (1), 23. 10.1186/s12014-023-09412-1 PMC10258980 37308827

[B35] Keeler A. M. Zhan W. Ram S. Fitzgerald K. A. Gao G. (2025). The curious case of AAV immunology. Mol. Ther. 33 (5), 1946–1965. 10.1016/j.ymthe.2025.03.037 PMC12126790 40156190

[B36] Knudsen P. J. Dinarello C. A. Strom T. B. (1987). Glucocorticoids inhibit transcriptional and post-transcriptional expression of interleukin 1 in U937 cells. J. Immunol. 139 (12), 4129–4134. 10.4049/jimmunol.139.12.4129 2826581

[B37] Lee S. W. Tsou A. P. Chan H. Thomas J. Petrie K. Eugui E. M. (1988). Glucocorticoids selectively inhibit the transcription of the interleukin 1 beta gene and decrease the stability of interleukin 1 beta mRNA. Proc. Natl. Acad. Sci. U. S. A. 85 (4), 1204–1208. 10.1073/pnas.85.4.1204 3257575 PMC279735

[B38] Lee Y. I. Hart C. C. Henley-Beasley C. S. Herr J. S. Zerpa E. Barton E. R. (2026). Functional and structural pathologies in skeletal muscle of a rat model of Duchenne muscular dystrophy. Skelet. Muscle 16 (1), 17. 10.1186/s13395-026-00419-4 PMC13040700 41742301

[B39] Lek A. Atas E. Hesterlee S. E. Byrne B. J. Bonnemann C. G. (2023a). Meeting report: 2022 muscular dystrophy association summit on 'safety and challenges in gene transfer therapy. J. Neuromuscul. Dis. 10 (3), 327–336. 10.3233/JND-221639 36806515

[B40] Lek A. Wong B. Keeler A. Blackwood M. Ma K. Huang S. (2023b). Death after high-dose rAAV9 gene therapy in a patient with duchenne's muscular dystrophy. N. Engl. J. Med. 389 (13), 1203–1210. 10.1056/NEJMoa2307798 PMC11288170 37754285

[B41] Leon-Astudillo C. Salabarria S. M. Chadwick C. B. Zingariello C. D. Berthy J. Prascak J. (2026). Sirolimus for the treatment of steroid-refractory hepatotoxicity following AAV gene therapy in patients with Duchenne muscular dystrophy. J. Neuromuscul. Dis., 22143602261424971. 10.1177/22143602261424971 PMC13438973 41854298

[B42] Lin X. Zhou L. Zhong J. Zhong L. Zhang R. Kang T. (2022). RNA-Binding protein RBM28 can translocate from the nucleolus to the nucleoplasm to inhibit the transcriptional activity of p53. J. Biol. Chem. 298 (2), 101524. 10.1016/j.jbc.2021.101524 PMC8789582 34953860

[B43] Lutz A. K. Pfaender S. Incearap B. Ioannidis V. Ottonelli I. Fohr K. J. (2020). Autism-associated SHANK3 mutations impair maturation of neuromuscular junctions and striated muscles. Sci. Transl. Med. 12 (547), eaaz3267. 10.1126/scitranslmed.aaz3267 32522805

[B44] Martinez-Chacin R. C. Bodrug T. Bolhuis D. L. Kedziora K. M. Bonacci T. Ordureau A. (2020). Ubiquitin chain-elongating enzyme UBE2S activates the RING E3 ligase APC/C for substrate priming. Nat. Struct. Mol. Biol. 27 (6), 550–560. 10.1038/s41594-020-0424-6 32393902 PMC7293561

[B45] Maurizi N. Ammirati E. Silver E. Hong K. Bui Q. Argiro A. (2026). Incidence, timing, and clinical significance of adverse immune events after gene replacement therapy: a systematic review and meta-analysis. Mol. Ther. 34 (3), 1340–1351. 10.1016/j.ymthe.2026.01.004 PMC12974193 41520173

[B46] Mendell J. R. Sahenk Z. Lehman K. Nease C. Lowes L. P. Miller N. F. (2020). Assessment of systemic delivery of rAAVrh74.MHCK7.micro-dystrophin in children with Duchenne muscular dystrophy: a nonrandomized controlled trial. JAMA Neurol. 77 (9), 1122–1131. 10.1001/jamaneurol.2020.1484 32539076 PMC7296461

[B47] Mendell J. R. Sahenk Z. Lehman K. J. Lowes L. P. Reash N. F. Iammarino M. A. (2024). Long-term safety and functional outcomes of delandistrogene moxeparvovec gene therapy in patients with Duchenne muscular dystrophy: a phase 1/2a nonrandomized trial. Muscle Nerve 69 (1), 93–98. 10.1002/mus.27955 37577753

[B48] Mendell J. R. Muntoni F. McDonald C. M. Mercuri E. M. Ciafaloni E. Komaki H. (2025). AAV gene therapy for Duchenne muscular dystrophy: the EMBARK phase 3 randomized trial. Nat. Med. 31 (1), 332–341. 10.1038/s41591-024-03304-z PMC11750718 39385046

[B49] Mendell J. R. Sahenk Z. Lowes L. P. Iammarino M. A. Alfano L. N. McDonald C. M. (2026). Five-year outcomes with delandistrogene moxeparvovec in patients with Duchenne muscular dystrophy: a phase 1/2a study. Muscle Nerve 74 (2), 397–403. 10.1002/mus.70276 PMC13332556 42237476

[B50] Muntoni F. Nascimento A. Shin J. Guglieri M. Stettner G. M. Veerapandiyan A. (2026). Safety and efficacy of fordadistrogene movaparvovec in ambulatory participants with Duchenne muscular dystrophy (CIFFREO): a phase 3, double-blind, randomised, placebo-controlled study. Lancet Neurology 25 (3), 245–255. 10.1016/s1474-4422(26)00036-0 41722591

[B51] Petrof B. J. Shrager J. B. Stedman H. H. Kelly A. M. Sweeney H. L. (1993). Dystrophin protects the sarcolemma from stresses developed during muscle contraction. Proc. Natl. Acad. Sci. U. S. A. 90 (8), 3710–3714. 10.1073/pnas.90.8.3710 8475120 PMC46371

[B52] Polge C. Attaix D. Taillandier D. (2015). Role of E2-Ub-conjugating enzymes during skeletal muscle atrophy. Front. Physiol. 6, 59. 10.3389/fphys.2015.00059 25805999 PMC4354305

[B53] Potter R. A. Moeller I. H. Khan S. Haegel H. Hollenstein A. Steiner G. (2025). Immunologic investigations into transgene directed immune-mediated myositis following delandistrogene moxeparvovec gene therapy. Sci. Rep. 15 (1), 4. 10.1038/s41598-024-84077-w PMC11696689 39747998

[B54] Rana J. Herzog R. W. Munoz-Melero M. Yamada K. Kumar S. R. P. Lam A. K. (2024). B cell focused transient immune suppression protocol for efficient AAV readministration to the liver. Mol. Ther. Methods Clin. Dev. 32 (1), 101216. 10.1016/j.omtm.2024.101216 PMC10911854 38440160

[B55] Saha M. Bhake R. Meyers C. A. Coleman K. Trivedi P. D. Gallman M. (2026). Enhancing AAV9-UFmuDys1 gene therapy efficacy through immunosuppression in mice with pre-existing immunity and enabling redosing strategies for Duchenne muscular dystrophy. Hum. Gene Ther. 37 (1-2), 17–32. 10.1177/10430342251385586 PMC13548098 41167625

[B56] Salabarria S. M. Corti M. Coleman K. E. Wichman M. B. Berthy J. A. D'Souza P. (2024). Thrombotic microangiopathy following systemic AAV administration is dependent on anti-capsid antibodies. J. Clin. Invest. 134 (1), e173510. 10.1172/JCI173510 PMC10760971 37988172

[B57] Schoggins J. W. Rice C. M. (2011). Interferon-stimulated genes and their antiviral effector functions. Curr. Opin. Virol. 1 (6), 519–525. 10.1016/j.coviro.2011.10.008 22328912 PMC3274382

[B58] Staeheli P. Grob R. Meier E. Sutcliffe J. G. Haller O. (1988). Influenza virus-susceptible mice carry Mx genes with a large deletion or a nonsense mutation. Mol. Cell Biol. 8 (10), 4518–4523. 10.1128/mcb.8.10.4518-4523.1988 2903437 PMC365527

[B59] Swart G. W. (2002). Activated leukocyte cell adhesion molecule (CD166/ALCAM): developmental and mechanistic aspects of cell clustering and cell migration. Eur. J. Cell Biol. 81 (6), 313–321. 10.1078/0171-9335-00256 12113472

[B60] Tereshko L. Zhao X. Gagnon J. Lin T. Ewald T. Wang Y. (2023). A novel method for quantitation of AAV genome integrity using duplex digital PCR. PLoS One 18 (12), e0293277. 10.1371/journal.pone.0293277 PMC10721069 38096204

[B61] van de Bovenkamp F. S. Dijkstra D. J. van Kooten C. Gelderman K. A. Trouw L. A. (2021). Circulating C1q levels in health and disease, more than just a biomarker. Mol. Immunol. 140, 206–216. 10.1016/j.molimm.2021.10.010 34735869

[B62] Willcocks R. J. Forbes S. C. Walter G. A. Sweeney L. Rodino-Klapac L. R. Mendell J. R. (2021). Assessment of rAAVrh.74.MHCK7.micro-dystrophin gene therapy using magnetic resonance imaging in children with Duchenne muscular dystrophy. JAMA Netw. Open 4 (1), e2031851. 10.1001/jamanetworkopen.2020.31851 PMC7783546 33394000

[B63] Xicluna R. Schwalie P. C. Bell E. Von Tell D. Steiner G. Seeger E. (2025). Innate immune response to AAV-Based gene therapy vectors: mechanisms of complement activation and cytokine release. Mol. Ther. Methods Clin. Dev. 33 (3), 101551. 10.1016/j.omtm.2025.101551 PMC12409308 40917687

[B64] Yuan R. Luo X. Liang Z. Cai S. Zhao Y. Zhu Q. (2024). UBE2C promotes myoblast differentiation and skeletal muscle regeneration through the akt signaling pathway. Acta Biochim. Biophys. Sin. (Shanghai) 56 (7), 1065–1071. 10.3724/abbs.2024062 PMC11322864 38690615

