# Supplementary material for: Systemic responses to intravenous rAAV administration in boys with Duchenne muscular dystrophy
Source: Front Mol Med. 2026 Sep 23;6:1953867. doi: 10.3389/fmmed.2026.1953867 (PMC13641740; doi:10.3389/fmmed.2026.1953867)
Supplement: Supplementary file 1 [file DataSheet1.pdf]

SUPPLEMENTAL MATERIALS

SUPPLEMENTAL TABLES

Table S1. Participant plasma and urine vector genome and protein content

Participant	Timepoint	Plasma Vector Genome Content (vg/mL)	Urine Vector Genome Content (vg/mL)	Plasma Vector Protein Content (vp/mL)	Urine Vector Protein Content (vp/mL)
1	Pre-Tx	n/a	<LLOQ	<LOD	<LOD
	Day 1	1.18E+12	<LLOQ	6.49E+11	<LOD
	Day 7	6.27E+08	<LLOQ	<LOD	<LOD
2	Pre-Tx	<LLOQ	<LLOQ	<LOD	<LOD
	Day 1	1.33E+12	4.90E+05	6.19E+11	<LOD
	Day 7	9.97E+08	<LLOQ	<LOD	<LOD
3	Pre-Tx	<LLOQ	<LLOQ	<LOD	<LOD
	Day 1	1.07E+12	5.97E+06	5.71E+11	<LOD
	Day 7	1.26E+09	1.17E+06	<LOD	<LOD
4	Pre-Tx	<LLOQ	n/a	<LOD	<LOD
	Day 1	9.70E+11	<LLOQ	4.99E+11	<LOD
	Day 7	5.56E+08	<LLOQ	<LOD	<LOD

The table displays the individual participant vector genome (vg/mL) and estimated vector plasma (vp/mL) content for both plasma and urine samples underlying the data presented in Figure 2 at baseline (Pre-Tx), Day 1, and Day 7 following treatment with 2×10^{14} vg/kg of AAVrh74.MHCK7.micro-dystrophin. Samples less than the limit of quantification (<LLOQ) for dPCR are shown as <LLOQ. Samples less than the limit of detection (<LOD) for immunoblotting are shown as <LOD.

Table S2. Top targets by significance in SomaScan

Target	Sequence ID	F Ratio	PValue	Logworth	FDR PValue	FDR Logworth	Effect Size
MX1	seq.17460.51	79.2915	1.60E-17	16.8072	8.24E-14	13.0843	0.96795
C3d	seq.5803.24	40.3031	1.10E-13	12.9616	2.89E-10	9.5396	2.24284
HMOX1	seq.17398.55	35.311	5.80E-13	12.2331	1.03E-09	8.9872	0.94596
MARCKSL1	seq.19175.18	28.1088	1.00E-11	10.9999	1.32E-08	7.879	2.97724
LST1	seq.9531.24	22.2985	1.60E-10	9.7838	1.74E-07	6.7598	2.63719
C1QTNF1	seq.6304.8	21.7829	2.20E-10	9.6632	1.91E-07	6.7184	2.94033
MAT2B	seq.18392.19	18.3127	1.65E-09	8.7833	1.24E-06	5.9054	2.01136
MPO	seq.2580.83	15.8373	8.55E-09	8.0681	5.65E-06	5.2483	3.41805
IGFBP7	seq.3320.49	15.5033	1.08E-08	7.9649	6.37E-06	5.1962	0.85378
EZR	seq.3320.49	15.3555	1.21E-08	7.9187	6.37E-06	5.1958	1.63559
NAGK	seq.3894.15	14.8822	1.70E-08	7.7683	8.19E-06	5.0867	0.8974
ACP5	seq.3232.28	14.5405	2.20E-08	7.6574	9.69E-06	5.0136	0.93751
TNFAIP8	seq.12563.2	13.9831	3.37E-08	7.4721	1.30E-05	4.8833	1.95769
IL6ST	seq.2620.4	13.9478	3.47E-08	7.4601	1.30E-05	4.8833	0.78398
DUSP3	seq.3480.7	13.8395	3.77E-08	7.4234	1.33E-05	4.8765	1.73061
ANG	seq.4874.3	13.0831	6.91E-08	7.1605	2.28E-05	4.6417	0.68125
LRRC15	seq.6557.50	12.9817	7.51E-08	7.1244	2.33E-05	4.6319	0.62713
EPHA4	seq.16288.17	12.4858	1.14E-07	6.9448	3.33E-05	4.4771	0.95651
COL28A1	seq.10702.1	12.2914	1.34E-07	6.8729	3.73E-05	4.4287	0.77444
DVL2	seq.13575.40	12.1965	1.45E-07	6.8375	3.84E-05	4.4156	1.1128
GRN	seq.4992.49	11.9632	1.78E-07	6.7496	4.23E-05	4.3737	1.6052
FAP	seq.5029.3	12.0199	1.69E-07	6.7711	4.23E-05	4.3737	0.63972
TNXB	seq.5698.60	11.9244	1.84E-07	6.7349	4.23E-05	4.3737	0.63343
EIF4H	seq.5885.55	11.8545	1.96E-07	6.7083	4.31E-05	4.3655	1.36086
VAT1	seq.18175.65	11.7927	2.07E-07	6.6846	4.37E-05	4.3596	1.61995
PLXND1	seq.19561.216	11.6258	2.40E-07	6.6203	4.87E-05	4.3123	0.71722
TFRC	seq.6895.1	11.4162	2.89E-07	6.5386	5.66E-05	4.247	2.13151
CRHBP	seq.6039.24	11.2813	3.27E-07	6.4854	6.17E-05	4.2096	1.06576
MFAP2	seq.9294.45	10.9474	4.45E-07	6.3518	8.10E-05	4.0912	0.67323
IL1R1	seq.2991.9	10.8696	4.78E-07	6.3202	8.43E-05	4.0744	1.56163
DIABLO	seq.3122.6	10.6664	5.79E-07	6.2371	9.87E-05	4.0055	0.73321
SEPW1	seq.18310.26	10.5748	6.32E-07	6.1992	1.04E-04	3.9814	1.36543
KAL1	seq.6603.18	10.3012	8.23E-07	6.0848	0.000132	3.8803	0.81958
CDON	seq.4541.49	10.2668	8.51E-07	6.0703	1.32E-04	3.8788	0.69151
IFIT3	seq.13642.90	10.1421	9.61E-07	6.0173	1.41E-04	3.8506	1.10672
TLR5	seq.18935.14	10.1614	9.43E-07	6.0255	1.41E-04	3.8506	0.97935
PCOLCE	seq.11237.49	9.98305	1.12E-06	5.949	1.61E-04	3.7943	0.64584

OLFML3	seq.8660.5	9.7887	1.37E-06	5.8646	1.90E-04	3.7214	0.78289
NUCB1	seq.10451.11	9.50799	1.82E-06	5.7407	2.40E-04	3.6198	0.87041
RET	seq.3220.40	9.51412	1.81E-06	5.7434	2.40E-04	3.6198	0.72069
PRND	seq.18231.147	9.4368	1.96E-06	5.7088	2.52E-04	3.5987	1.25598
EPHB6	seq.5078.82	9.39614	2.04E-06	5.6906	2.57E-04	3.5909	0.78219
EPHB4	seq.15530.33	9.36601	2.10E-06	5.677	2.59E-04	3.5875	0.76219
HIST3H2BB	seq.18823.52	9.2625	2.34E-06	5.6302	2.81E-04	3.5507	1.97518
DUT	seq.9995.6	9.20406	2.49E-06	5.6036	2.92E-04	3.5339	1.85458
IL19	seq.3035.80	9.15899	2.61E-06	5.5831	3.00E-04	3.5229	0.96473
COL6A3	seq.10511.10	9.0821	2.83E-06	5.5478	3.02E-04	3.5199	0.70341
PAPSS1	seq.14007.22	9.10702	2.76E-06	5.5592	3.02E-04	3.5199	1.44308
CDH5	seq.2819.23	9.10235	2.77E-06	5.5571	3.02E-04	3.5199	0.70491
ADAMTSL2	seq.6379.62	9.07354	2.86E-06	5.5439	3.02E-04	3.5199	1.1239
LCP1	seq.17231.1	8.97107	3.19E-06	5.4965	3.30E-04	3.4811	0.9198
B4GALT6	seq.10832.24	8.9328	3.32E-06	5.4788	3.37E-04	3.4718	1.01922
CTHRC1	seq.15467.10	8.90678	3.41E-06	5.4667	3.40E-04	3.468	0.65412
CD200R1	seq.5103.30	8.84019	3.67E-06	5.4356	3.59E-04	3.445	1.33376
LRRC4C	seq.9369.174	8.77791	3.92E-06	5.4063	3.77E-04	3.4237	0.92636
DYNLRB2	seq.17827.53	8.73778	4.10E-06	5.3874	0.00038	3.4203	0.93592
FAM3B	seq.9177.6	8.7385	4.09E-06	5.3878	3.80E-04	3.4203	0.84941
CRABP2	seq.11696.7	8.6414	4.55E-06	5.3418	4.15E-04	3.3823	0.67626
TXNL4B	seq.15316.262	8.60032	4.76E-06	5.3223	4.26E-04	3.3701	1.58137
ROR1	seq.2590.69	8.57782	4.88E-06	5.3115	4.30E-04	3.3667	0.71317
C1QA C1QB C1QC	seq.2753.2	8.54055	5.08E-06	5.2937	4.40E-04	3.3561	1.07757
COL1A1	seq.11140.56	8.46953	5.50E-06	5.2596	4.57E-04	3.3405	0.65843
RBM28	seq.11927.3	8.49206	5.36E-06	5.2705	4.57E-04	3.3405	1.63884
NPTN	seq.7194.36	8.46476	5.53E-06	5.2573	0.000457	3.3405	0.81286
UNC5B	seq.7776.20	8.43516	5.71E-06	5.2431	0.000465	3.333	0.72338
AFM	seq.4763.31	8.35489	6.25E-06	5.2042	5.00E-04	3.3008	1.10496
SHANK3	seq.13242.134	8.31771	6.51E-06	5.1861	5.14E-04	3.2892	1.64601
NFKBID	seq.19123.6	8.30397	6.62E-06	5.1794	5.14E-04	3.289	1.15427
FLRT2	seq.13122.19	8.12371	8.11E-06	5.0909	5.95E-04	3.2253	0.72214
THTPA	seq.13595.20	8.1399	7.96E-06	5.0989	5.95E-04	3.2253	1.00374
OLFM2	seq.19377.14	8.15239	7.85E-06	5.1051	5.95E-04	3.2253	0.66265
DYNLRB1	seq.3845.51	8.12652	8.08E-06	5.0923	0.000595	3.2253	0.98969
RAB26	seq.6997.32	8.08967	8.43E-06	5.0741	6.10E-04	3.2145	1.32289
CLEC3B	seq.5701.81	8.05198	8.80E-06	5.0554	6.29E-04	3.2017	0.64319
B3GALT2	seq.11638.42	8.0277	1.00E-05	5.0433	6.31E-04	3.1999	1.09314
FTL	seq.15324.58	8.01387	1.00E-05	5.0364	6.31E-04	3.1999	0.83749

COLEC11	seq.4430.44	8.01839	1.00E-05	5.0387	0.000631	3.1999	0.97266
MXRA8	seq.10521.10	7.99195	1.00E-05	5.0255	6.39E-04	3.1946	0.68901
GCH1	seq.11185.145	7.95011	1.00E-05	5.0046	0.000656	3.183	1.99781
ALOX15B	seq.12422.143	7.92661	1.00E-05	4.9928	0.000656	3.183	2.82563
ABHD10	seq.17370.186	7.94101	1.00E-05	5	6.56E-04	3.183	1.11636
RPRD1B	seq.19126.4	7.91492	1.00E-05	4.9869	6.56E-04	3.183	1.40808
FTH1 FTL	seq.5934.1	7.91508	1.00E-05	4.987	6.56E-04	3.183	0.83603
CHAD	seq.13460.4	7.88055	1.00E-05	4.9696	6.75E-04	3.1709	0.61229
ALCAM	seq.5451.1	7.81586	1.16E-05	4.9369	7.19E-04	3.1434	1.15535
TSSK1B	seq.11179.7	7.78244	1.20E-05	4.92	7.39E-04	3.1315	1.08679
BLNK	seq.19225.11	7.76018	1.23E-05	4.9087	7.49E-04	3.1252	0.78981
NEK7	seq.12703.6	7.71642	1.30E-05	4.8864	7.71E-04	3.1128	1.23751
IL20RA	seq.5085.18	7.71692	1.30E-05	4.8866	7.71E-04	3.1128	1.79356
FGL1	seq.5581.28	7.67419	1.37E-05	4.8648	8.02E-04	3.0961	1.34728
SPON2	seq.8099.42	7.65834	1.39E-05	4.8567	8.08E-04	3.0927	0.87016
CTSH	seq.8465.52	7.61158	1.47E-05	4.8326	8.35E-04	3.0782	1.28474
TFPI2	seq.9233.71	7.61692	1.46E-05	4.8354	0.000835	3.0782	0.81572
HIST2H2BE	seq.14143.8	7.5357	1.61E-05	4.7935	0.000904	3.0436	1.09845
Unknown	seq.4666.232	7.52104	1.64E-05	4.7859	0.000911	3.0406	0.90481
SULT4A1	seq.8357.43	7.50667	1.67E-05	4.7784	0.000917	3.0377	1.6776
ARL5B	seq.17404.5	7.45438	1.77E-05	4.7512	0.000962	3.017	1.25694
C1S	seq.8840.61	7.44962	1.78E-05	4.7487	0.000962	3.017	1.10275
INSR	seq.3448.13	7.41218	1.87E-05	4.7292	0.000996	3.0019	1.02489

The table displays the top differentially expressed targets identified by a higher scrutiny threshold (FDR p-value < 0.001). These are ranked by order of significance, with the most significantly changed targets displayed first. Target names are shown, along with the statistical parameters from repeated measures ANOVA. These include the F Ratio, p-value, Logworth [$-\log_{10}(p)$], the FDR-adjusted p-value, the FDR Logworth [$-\log_{10}(\text{FDR } p)$], and the associated effect size.

SUPPLEMENTAL FIGURES

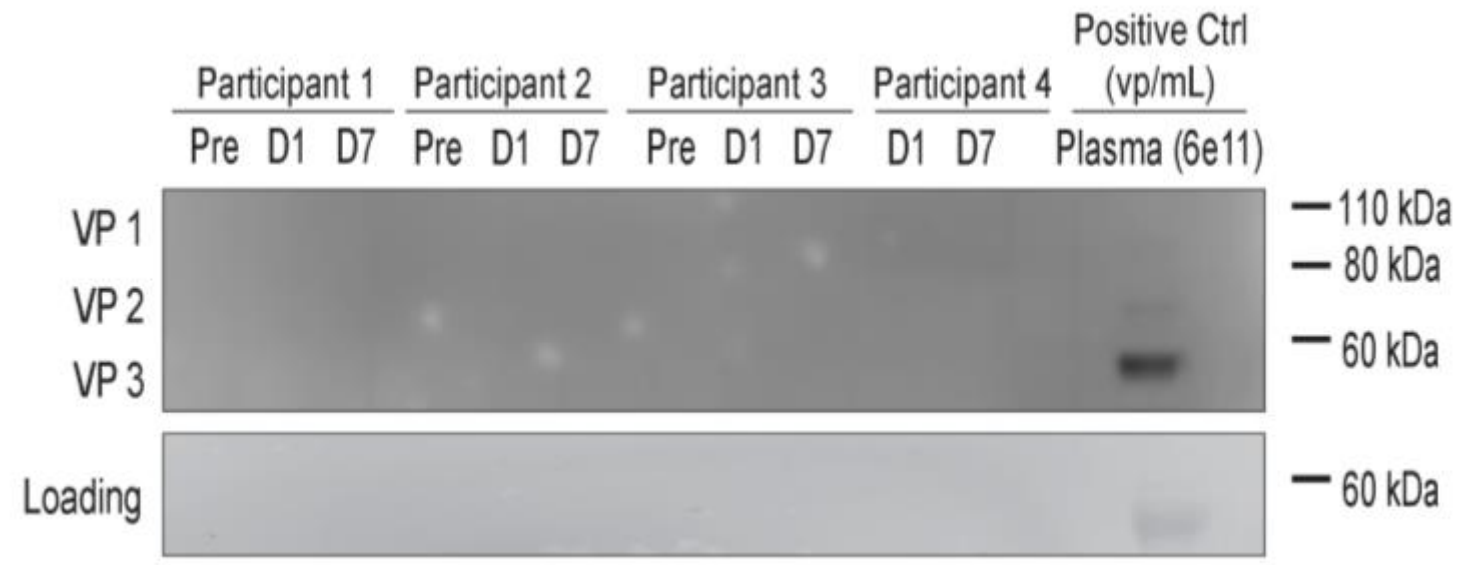

Figure S1. Immunoblot of AAV capsid proteins in participant urine. AAV capsid proteins (VP1, VP2, VP3) were detected by immunoblotting in urine samples from Participant's 1-4 collected at baseline (Pre), Day 1 (D1), and Day 7 (D7) following treatment with 2×10^{14} vg/kg of AAVrh74.MHCK7.micro-dystrophin. A positive control plasma sample containing an estimated 6×10^{11} vp/mL was run alongside urine samples.

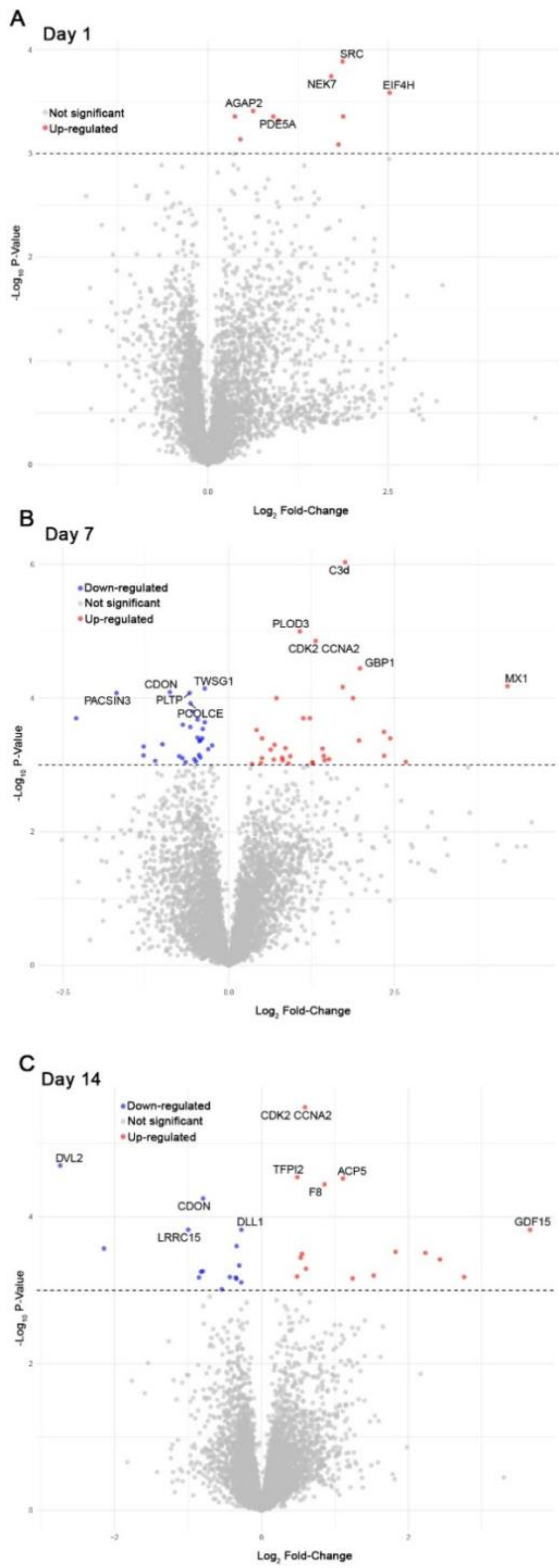

Figure S2. SomaScan volcano plots for Days 1, 7, and 14. Volcano plot of differentially expressed proteins showing fold-change of (**A**) Day 1, (**B**) Day 7, and (**C**) Day 14 plasma samples from pre-treatment baseline. Each point represents a single protein. The x-axis represents $\log_2$ fold change, indicating magnitude and direction of change. The y-axis represents statistical significance as $-\log_{10}$ p-value with the dashed line indicating p-value < 0.001 threshold, with positively and negatively significantly changed targets shown in red or blue, respectively.

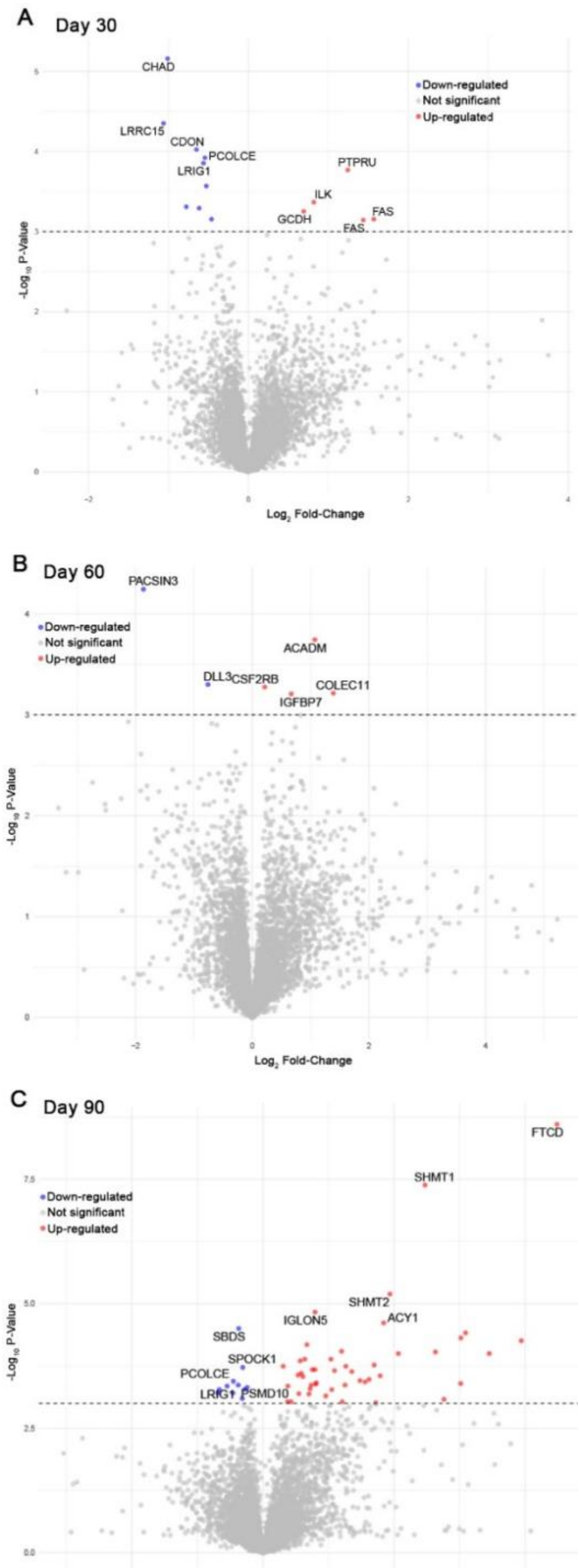

Figure S3. SomaScan volcano plots for Days 30, 60, and 90. Volcano plot of differentially expressed proteins showing fold-change of (**A**) Day 30, (**B**) Day 60, and (**C**) Day 90 plasma samples from pre-treatment baseline. Each point represents a single protein. The x-axis represents $\log_2$ fold change, indicating magnitude and direction of change. The y-axis represents statistical significance as $-\log_{10}$ p-value with the dashed line indicating p-value < 0.001 threshold, with positively and negatively significantly changed targets shown in red or blue, respectively.

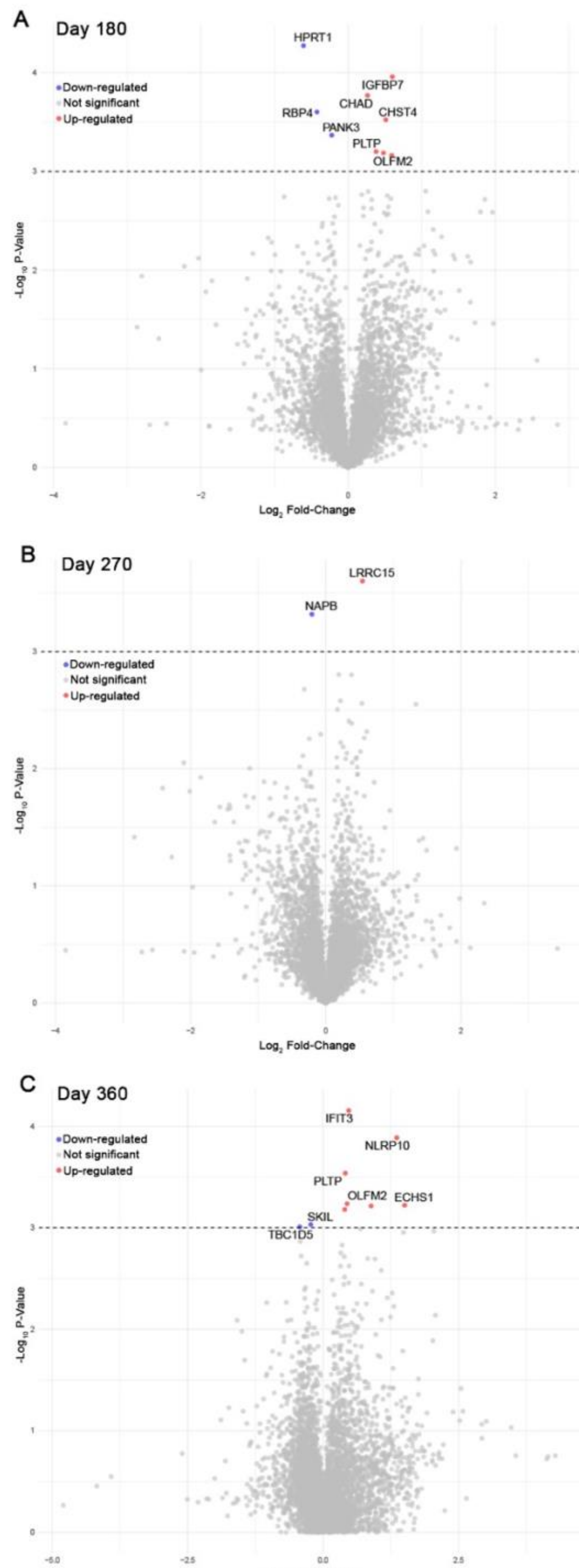

Figure S4. SomaScan volcano plots for Days 180, 270, and 360. Volcano plot of differentially expressed proteins showing fold-change of (**A**) Day 180, (**B**) Day 270, and (**C**) Day 360 plasma samples from pre-treatment baseline. Each point represents a single protein. The x-axis represents $\log_2$ fold change, indicating magnitude and direction of change. The y-axis represents statistical significance as $-\log_{10}$ p-value with the dashed line indicating p-value < 0.001 threshold, with positively and negatively significantly changed targets shown in red or blue, respectively.

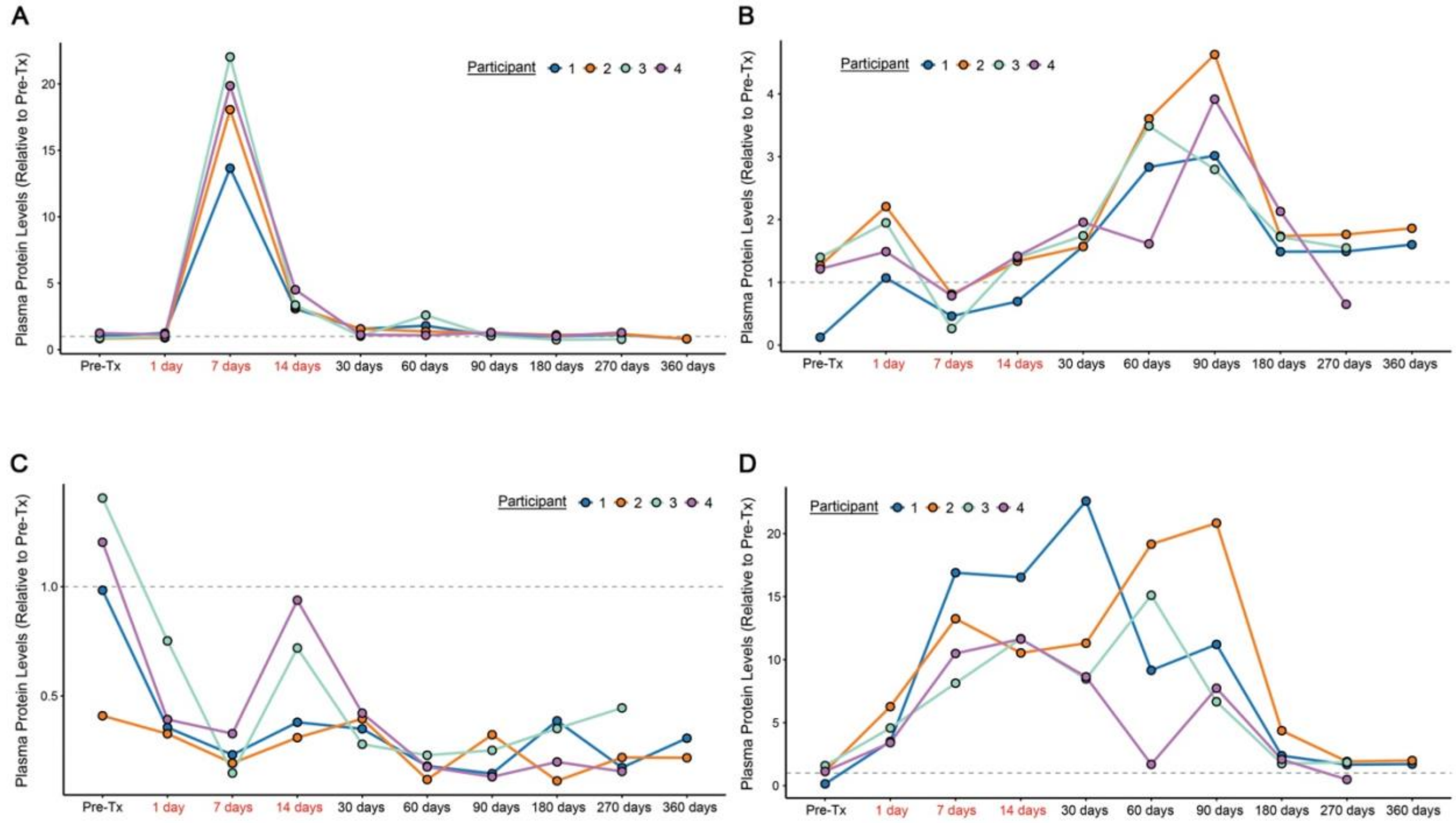

Figure S5. Individual participant trajectories of SomaScan proteomic markers identified in rAAV-treated DMD plasma. Individual participant trajectories underlying immune-associated (Figure 4), muscle-associated (Figure 5), and organ injury (Figure 7) marker panels are shown, as measured by SomaScan aptamer-based proteomic profiling (n=4 for Pre-Tx to Day 270; n=2 for Day 360): (**A**) MX1 participant trajectories, (**B**) IL1R1 participant trajectories, (**C**) TNNI3 participant trajectories, (**D**) GDF15 participant trajectories. Plasma protein levels are expressed relative to the averaged participant baseline (Pre-Tx) values, with lines connecting the individual participant values across timepoints as indicated in the legend by color. Timepoints indicated by red font indicate interval of daily steroid regimen.
